# Shifting transcriptional machinery is required for long-term memory maintenance and modification in *Drosophila* mushroom bodies

**DOI:** 10.1038/ncomms13471

**Published:** 2016-11-14

**Authors:** Yukinori Hirano, Kunio Ihara, Tomoko Masuda, Takuya Yamamoto, Ikuko Iwata, Aya Takahashi, Hiroko Awata, Naosuke Nakamura, Mai Takakura, Yusuke Suzuki, Junjiro Horiuchi, Hiroyuki Okuno, Minoru Saitoe

**Affiliations:** 1 SK Project, Medical Innovation Center, Kyoto University Graduate School of Medicine, 53 Shogoin Kawahara-cho, Sakyo-ku, Kyoto 606-8507, Japan; 2 Japan Science and Technology Agency, PRESTO, 4-4-8 Honcho, Kawaguchi, Saitama 332-0012, Japan; 3 Center of Gene Research, Nagoya University, Huro-cho, Chikusa-ku, Nagoya 464-8602, Japan; 4 Tokyo Metropolitan Institute of Medical Science, 2-1-6 Kamikitazawa, Setagaya, Tokyo 156-0057, Japan; 5 Center for iPS Cell Research and Application, Department of Reprogramming Science, Kyoto University, 53 Shogoin Kawahara-cho, Sakyo-ku, Kyoto, Kyoto 606-8507, Japan; 6 Institute for Integrated Cell-Material Sciences (WPI-iCeMS), Kyoto University, 53 Shogoin Kawahara-cho, Sakyo-ku, Kyoto, Kyoto 606-8507, Japan; 7 AMED-CREST, AMED 1-7-1 Otemach, Chiyodaku, Tokyo 100-0004, Japan; 8 Kyoto Sangyo University, Motoyama, Kamigamo, Kita-ku, Kyoto City 603-8555, Japan

## Abstract

Accumulating evidence suggests that transcriptional regulation is required for maintenance of long-term memories (LTMs). Here we characterize global transcriptional and epigenetic changes that occur during LTM storage in the *Drosophila* mushroom bodies (MBs), structures important for memory. Although LTM formation requires the CREB transcription factor and its coactivator, CBP, subsequent early maintenance requires CREB and a different coactivator, CRTC. Late maintenance becomes CREB independent and instead requires the transcription factor Bx. Bx expression initially depends on CREB/CRTC activity, but later becomes CREB/CRTC independent. The timing of the CREB/CRTC early maintenance phase correlates with the time window for LTM extinction and we identify different subsets of CREB/CRTC target genes that are required for memory maintenance and extinction. Furthermore, we find that prolonging CREB/CRTC-dependent transcription extends the time window for LTM extinction. Our results demonstrate the dynamic nature of stored memory and its regulation by shifting transcription systems in the MBs.

## Abstract (web summary)

Transcriptional regulation is necessary for maintaining long-term memories (LTM) but the mechanistic details are not completely defined. Here the authors identify transcriptional machinery and histone modifiers required for LTM maintenance in *Drosophila* and show that transcriptional regulation for LTM maintenance is distinct from that for LTM formation.

New experiences can be consolidated in the brain as long-term memories (LTMs), which are stored in the brain for subsequent retrieval. During LTM formation, the transcription factor CREB (Ca^2+^/cAMP-responsive element-binding protein) plays a critical role by promoting gene expression^1^,^2^ in concert with epigenetic factors such as histone acetyl transferases (HATs) and DNA methylases^3^. In particular, CREB forms a complex with the HAT, CREB-binding protein (CBP), required for LTM formation^4^,^5^,^6^. Although the importance of the HAT activity of CBP in LTM has been debated^7^,^8^, dynamic alteration in histone acetylation after leaning^9^,^10^ and suppression of LTM by counteracting protein to HAT, histone deacetylase 2 (ref. 11), supports a model where histone acetylation is important in LTM formation.

Recent studies suggest that changes in transcription are also actively involved during memory storage^12^,^13^. In *Aplysia*, LTM maintenance is correlated with DNA methylation at the promoter region of CREB2, a transcriptional repressor, which inhibits activity of the CREB1 activator^14^. In rat cortical neurons, DNA methylation at *calcineurin*, a memory suppressor gene^15^, is elevated during learning and the persistence of this methylation is critical for LTM maintenance^12^. In mice, a recent study reported alterations in DNA methylation coupled to gene expression after learning and, importantly, this study demonstrated differences in gene expression between LTM formation and maintenance^16^. These results suggest that epigenetic modifications are also important for LTM maintenance, and that transcriptional programmes in LTM formation and maintenance are distinct processes.

*Drosophila* forms aversive olfactory associations if they are exposed to an odour (the conditioned stimulus or CS) paired with electric shocks (the unconditioned stimulus). Flies form LTM of this association if they are trained repeatedly in this association with rest intervals interspersed between trainings (spaced training)^17^. In *Drosophila*, the mushroom bodies (MBs) are brain structures critical for olfactory memory^18^,^19^,^20^. Here we identify transcriptional machinery and histone modifiers required for LTM maintenance in *Drosophila* MBs. We found that transcriptional regulation for LTM maintenance is distinct from that for LTM formation. Although formation depends on CREB and CBP, subsequent maintenance, up to 4 days after formation, is dependent on CREB and another coactivator, CRTC (CREB-regulated transcription coactivator). Later maintenance no longer requires CREB/CRTC activity, but requires activity of another transcription factor, Bx. We finally demonstrate that this shift of transcription system is critical for restricting modification of LTM to early stage of LTM maintenance. Our results suggest that memory maintenance is a dynamic, shifting process with distinct temporal functions.

## Results

### Shifting CREB-related functions in LTM

As CREB plays a pivotal role in LTM formation in *Drosophila* and other species^21^, we first tested whether CREB activity is still required for LTM maintenance. In *Drosophila*, transcriptional activity of the fly CREB homologue dCREB2 is inhibited by dCREB2-b, a repressor isoform^22^. To suppress activity of dCREB2 during LTM storage, we overexpressed a *CREB2-b* transgene from the MB247-switch (MBsw) driver, where expression of *CREB2-b* can be induced in the MBs by feeding flies RU486 (RU; ref. 23).

**Figure 1:**
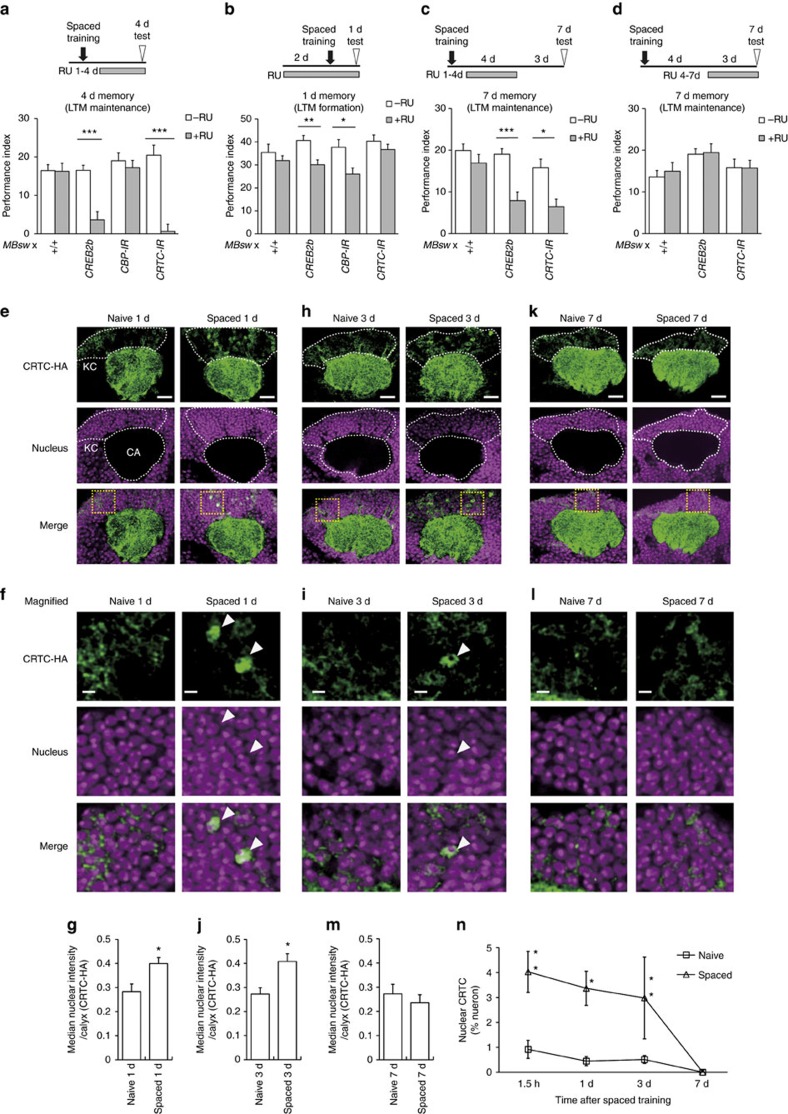
CREB/CRTC is required for LTM maintenance but not for LTM formation. (**a**–**d**) Knockdown of *CRTC* impairs 4-day LTM maintenance. Wild-type flies (+/+) or the flies carrying the indicated UAS transgenes were crossed with MBsw and their progeny were tested as shown to test 4-day LTM maintenance (**a**, from left to right, *n*=10, 10, 10, 10, 10, 10, 8 and 8), LTM formation (**b**, from left to right, *n*=7, 8, 8, 8, 8, 10, 8 and 8) and 7-day LTM maintenance (**c**, from left to right, *n*=10, 12, 10, 8, 8 and 8; **d**, from left to right, *n*=11, 10, 10, 8, 8 and 8)). *P<0.05, **P<0.01 and ***P<0.001 by Student's *t* test. (**e**–**n**) Subcellular localization of CRTC in the MBs from 1 to 3 days, but not 7 days after spaced training. *UAS-CRTC-HA* flies were crossed with a MB GAL driver *MB247*. CRTC-HA was stained using anti-HA antibodies (green) and the nuclei were stained by TO-PRO-3 iodide (magenta). The images are representative of four to five experimental replicates, which are quantified in **g**–**n**. (**e**,**h**,**k**) The MB cell bodies, Kenyon cells (KC) and the dendritic region calyx (CA) are indicated by white dotted lines. The naive control flies were sampled at the same age after eclosion as the spaced trained flies. Scale bars, 10 μm. (**f**,**i**,**l**) Magnified view of the yellow squared area in **e**,**h** and **k**, respectively. White arrow heads indicate nuclear translocation of CRTC. Scale bars, 2 μm. (**g**,**j**,**m**) Median CRTC-HA nuclear intensities are increased at 1 and 3 days, but not 7 days, after spaced training. The individual nuclear CRTC-HA signal intensities were normalized to those in the calyx. The median nuclear CRTC-HA signal intensities in each brain was obtained and the averaged medians were shown (from left to right in **g**–**m**, *n*=4, 4, 5, 4, 5 and 4). *P<0.05 by Mann–Whitney *U*-test. (**n**) Median number of the nuclei showing more CRTC-HA intensities than the calyx (naive from left to right, *n*=5, 4, 5 and 5; spaced training from left to right t, *n*=4, 4, 4 and 5). A two-way analysis of variance of the data indicates significant effect of time (*P*<0.001) and treatment (naive or spaced; *P*<0.001), but not in interaction between them (*P*>0.66). **P<0.01 by Bonferroni testing.

When *CREB2-b* expression was induced 1 day after spaced training, we found remarkable impairment in 4-day LTM (memory 4 days after spaced training; Fig. 1a). LTM formation requires the CREB coactivator CBP^4^,^5^,^6^,^24^. To test whether CBP is also required for LTM maintenance, we performed RNA interference (RNAi)-based knockdown of *CBP* (*CBP-IR*) using the MBsw driver^24^. Although knocking down *CBP* 2 days before spaced training significantly disrupted 1-day LTM (Fig. 1b), knocking down *CBP* from 1 to 4 days after training did not affect 4-day LTM (Fig. 1a). These results suggest that although transcriptional activity of CREB is still required, a different CREB coactivator may be recruited for LTM maintenance.

In addition to CBP, CRTC also regulates transcriptional activity of CREB^25^. As reported previously^24^, expression of *CRTC-IR* in the MBs did not affect 1-day LTM after spaced training (Fig. 1b)^24^. However, knockdown of CRTC from 1 to 4 days after training significantly disrupted 4-day LTM (Fig. 1a) and 7-day LTM (Fig. 1c). Furthermore, neither expressing *CREB2-b* nor *CRTC-IR* from 4 to 7 days after spaced training affected 7-day LTM (Fig. 1d). This suggests that CREB/CRTC activity in the MBs is required for LTM maintenance from 1 to 4 days after training, but not for maintenance from 4 to 7 days. Thus, a different transcription system may maintain LTM after 4 days.

The requirement of CREB/CRTC for maintenance of LTM 1 to 4 days after training is also supported by the localization of CRTC (Fig. 1e–n). In the inactive state, CRTC is phosphorylated and sequestered in the cytoplasm^24^,^25^. On activation, CRTC is dephosphorylated and translocates into the nucleus to promote CREB-dependent gene expression^26^,^27^. We observed nuclear translocation of CRTC at 1.5 h after spaced training (Supplementary Fig. 1a–c). This translocation was not observed after massed training (Supplementary Fig. 1a–c), repeated trainings without rest intervals, which produces only protein synthesis-independent anaesthesia-resistant memory^17^, indicating that nuclear translocation of CRTC is specific to LTM. CRTC still localized to the nuclei 1 day (Fig. 1e–g) and 3 days after spaced training (Fig. 1h–j), and returned to the cytoplasm by 7 days (Fig. 1k–m). CRTC accumulated in the nuclei in ∼4% of MB Kenyon cells after spaced training (Fig. 1n and Supplementary Fig. 1d), a number consistent with the population of MB neurons responding to an odour (6±5%)^28^. These observations support that translocation of CRTC is a molecular mechanism regulating 4-day LTM maintenance.

### Shifting HAT requirement in LTM formation and maintenance

**Figure 2:**
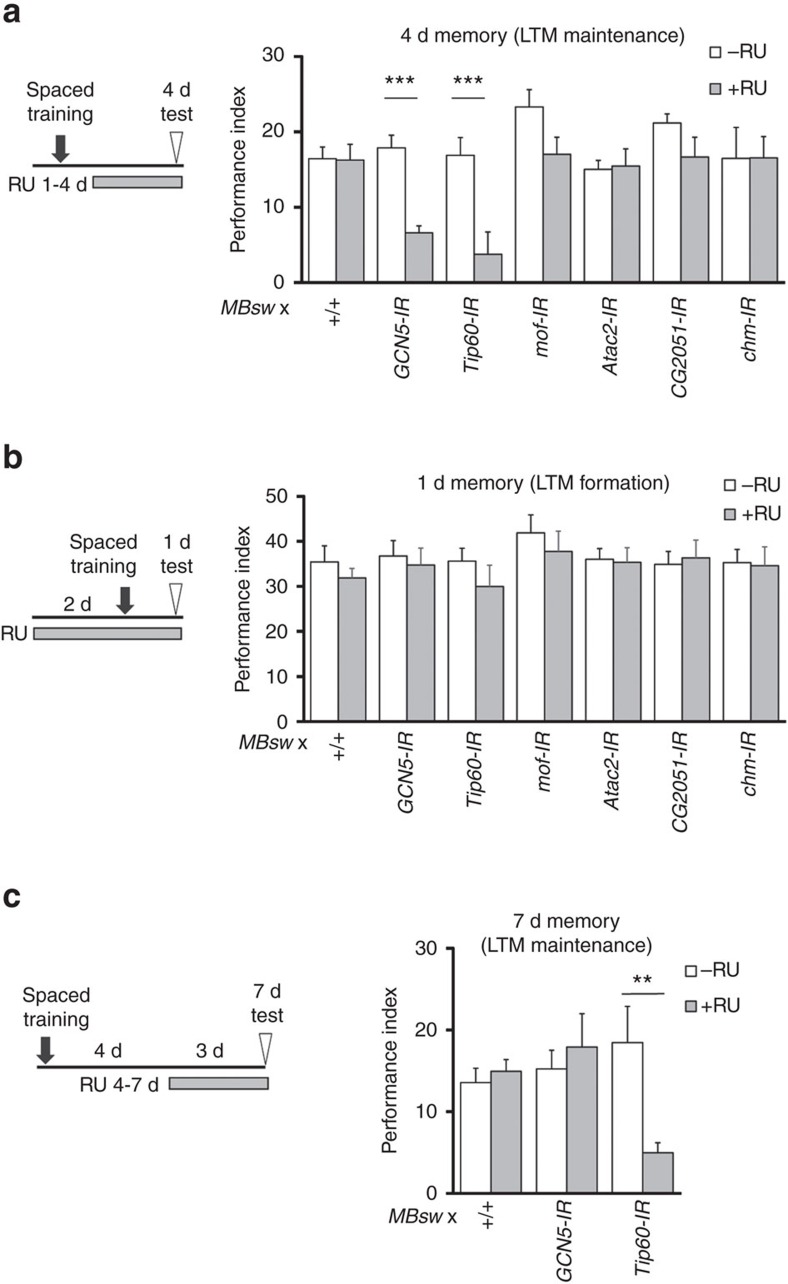
HATs, GCN5 and Tip60 are required for LTM maintenance. (**a**) Knockdown of *GCN5* and *Tip60* impairs 4-day LTM maintenance (from left to right, *n*=10, 10, 11, 11, 8, 7, 8, 8, 11, 12, 8, 9, 8 and 8). (**b**) Knockdown of *GCN5* and *Tip60* does not affect LTM formation (from left to right, *n*=7, 8, 8, 8, 8, 8, 8, 8, 7, 7, 8, 8, 8 and 8). (**c**) Knockdown of *Tip60*, but not *GCN5*, impairs 7-day LTM maintenance. Wild-type flies (+/+) or the flies carrying the indicated UAS transgenes were crossed with MBsw and their progeny were tested as shown (from left to right, *n*=11, 10, 10, 9, 7 and 8). *P<0.05, **P<0.01 and ***P<0.001 by Student's *t*-test.

As CBP is not involved in LTM maintenance, we postulated that other HATs may be involved. To identify these HATs, we inhibited expression of all known *Drosophila* HATs, including *GCN5* (ref. 29), *Tip60* (refs 30, 31), *mof*^32^, *Atac2* (ref. 33), *chm*^34^ and *CG2051* (inferred from sequence similarity; FlyBase, http://flybase.bio.indiana.edu/). We measured the efficiency of knockdown in all cases (Supplementary Fig. 2a–f) and tested the effects on LTM. Among these, knockdown of *GCN5* and *Tip60* from 1 to 4 days after spaced training significantly impaired 4-day LTM (Fig. 2a), whereas knockdown of these HATs did not affect 1-day LTM (Fig. 2b). These results further suggest that LTM formation and maintenance require distinct transcriptional machineries. Furthermore, knockdown of *Tip60* starting from 4 to 7 days after spaced training impaired 7-day LTM, whereas knockdown of *GCN5* did not (Fig. 2c). This indicates that 4-day LTM maintenance requires CREB/CRTC, GCN5 and Tip60, whereas 7-day LTM maintenance requires other transcriptional machinery including Tip60.

The MBs consist of different subpopulations of Kenyon cells, which innervate the α/β, α′/β′and γ lobes. MBsw targets most of these subpopulations, including α/β surface (α/βs), α/β core and γ main neurons (Supplementary Fig. 3a). To identify which subpopulation(s) are required for LTM maintenance, we next used split-GAL4 lines^35^ to target specific cell-types. Knocking down *CRTC* in α/βs neurons significantly impaired 4-day LTM, whereas knocking down *CRTC* in α/β core, γ main, α'/β' middle (α'/β' m) and α'/β' anterior posterior (α'/β' ap) did not (Supplementary Fig. 3d,e). One-day LTM was unaffected by knocking down *CRTC* in any of these cell types (Supplementary Fig. 3b,c). Unfortunately, split-GAL4 drivers cannot be regulated temporally and knockdown of *GCN5* and *Tip60* in most subpopulations impaired 1-day memory (Supplementary Fig. 3f,g). As acute knockdown of these genes using MBsw had no effect on 1-day memory (Fig. 2b), *GCN5* and *Tip60* are likely to be required during development for normal MB function. Thus, the α/βs neurons are the subpopulation that requires CRTC for 4-day LTM maintenance, which has been suggested as the neurons important for retrieval of aversive memory^36^.

### Histone acetylation emerged after LTM formation

**Figure 3:**
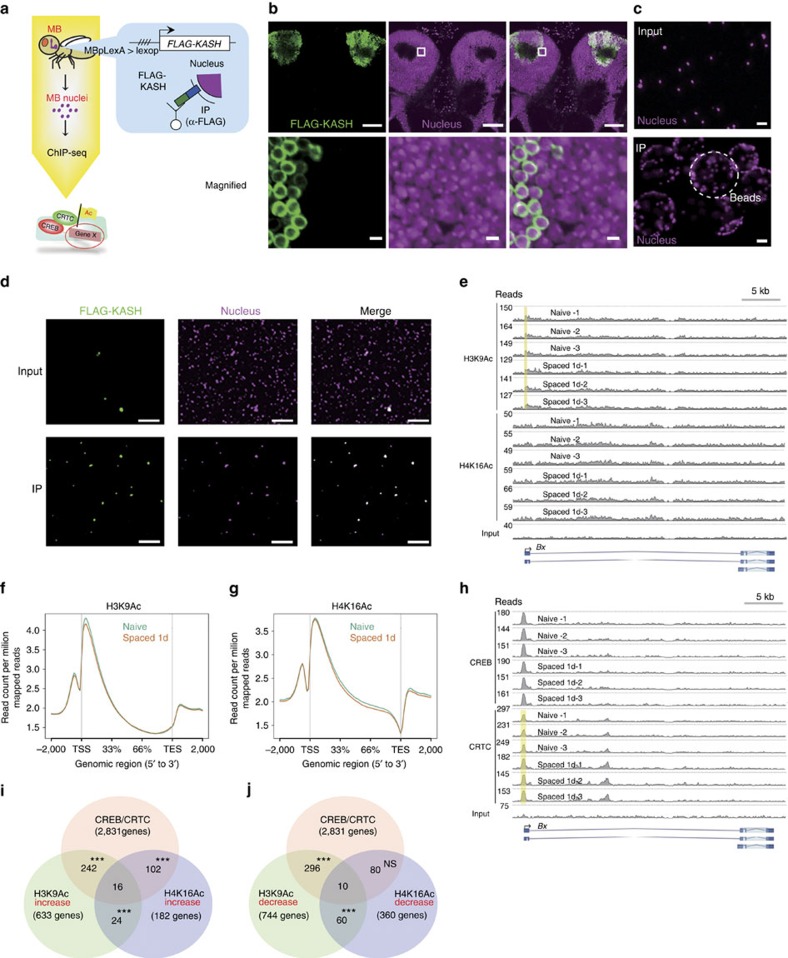
Identification of CREB/CRTC and histone acetylation target genes in the MBs. (**a**) Schematic diagram of the experimental design. (**b**) Subcellular localization of FLAG-KASH in the MBs. FLAG-KASH was expressed by *MBp-LexA*. (upper) anti-FLAG staining (green) and nuclear staining by TO-PRO-3 iodide were shown (magenta). Scale bars, 50 μm. (lower) Magnified view of the white squared area in the upper panel. Scale bars, 2 μm. The images are representative of three experimental replicates. (**c**) Nuclear staining of immunoprecipitated MB nuclei. Upper: the heads were homogenized and the nuclei were stained propidium iodide (magenta). Lower: the nuclei were immunoprecipitated using anti-FLAG antibody-conjugated beads. The nuclei were stained by propidium iodide (magenta). Scale bars, 25 μm. The images are representative of four experimental replicates. (**d**) The immunoprecipitated nuclei expressed FLAG-KASH. The precipitated nuclei in **c** were released from the beads and stained with anti-FLAG antibody (green). The nuclei were stained by propidium iodide (magenta). Scale bars, 25 μm. The images are representative of three experimental replicates. (**e**,**h**) ChIP-seq signals at the region near *Bx*. Purified MB nuclei expressing FLAG-KASH and prepared from naive flies or flies 1 day after spaced training were subjected to ChIP-seq analysis, using anti-H3K9Ac (*n*=3), anti-H4K16 (*n*=3) (**e**), anti-CREB (*n*=3) and anti-CRTC (*n*=3) antibodies (**j**). The sequencing data of input DNA was shown at the bottom. Increases in histone acetylation and CREB/CRTC-binding sites identified in this study are highlighted by light-yellow vertical bars. The *y* axes show the number of the mapped reads and the upper limits were adjusted to the number of the total reads in each samples. The base line indicates zero read. (**f**,**g**) Aggregate gene plot of H3K9Ac (**f**) and H4K16Ac (**g**). The mapped reads of naive samples or spaced-trained samples were pooled (*n*=3 for each, shown in green and orange lines, respectively) and were summarized with respect to the distance from the TSS and the transcriptional end site (TES). (**i**,**j**) Venn diagram showing overlaps of CREB/CRTC-binding genes where CRTC binding is increased and the genes with increases (**i**) or decreases (**j**) in H3K9Ac and H4K16Ac in 500 bp vicinity to TSS. The non-random overlaps indicated by hypergeomic test were shown by asterisks. NS, not significant; ***P<0.001.

The requirements of GCN5 and Tip60 for LTM maintenance suggests that histone acetylation in the MBs is necessary for maintenance. To determine target acetylated genes in the MBs, we purified MB nuclei, using a method previously used to isolate nuclei in plants^37^ and mice^38^. A FLAG-tagged KASH (Klarsicht/ANC-1/Syne-1 homology) domain, which is inserted into the nuclear membrane with the FLAG-tag facing the cytoplasm^39^ was expressed in the MBs from a *MBp-LexA* driver^40^ (Fig. 3a). The *MBp-LexA* strongly labels the α/β and γ lobes, and weakly labels the α'/β' lobe (Supplementary Fig. 4a). Immunohistochemistry showed specific labelling of MB nuclei by FLAG-KASH (Fig. 3b). By immunoprecipitating with anti-FLAG antibody, FLAG-KASH expressing MB nuclei were prepared from whole brains (Fig. 3c,d) to an estimated purity of ∼90% (Supplementary Fig. 4b). MB nuclei prepared from naive flies or flies 1 day after spaced training were subjected to chromatin immunoprecipitation combined with high-throughput sequencing (ChIP-Seq) analysis. Read qualities were confirmed (Supplementary Data 1 and see Methods) and principal component analysis showed that the biological replicates were classified by their condition, naive or spaced trained (Supplementary Fig. 5).

GCN5 acetylates histone lysine residues including H3K9 (H3K9Ac)^29^ and Tip60 acetylates a different set of residues including H4K16 (H4K16Ac)^41^. We found the 1,013 and 423 regions showing significant increase in H3K9Ac and H4K16Ac, respectively, 1 day after spaced training (Fig. 3e, Supplementary Fig. 6a,b and Supplementary Data 3 and 4). Aggregate gene plot indicated accumulation of the mapped reads in the vicinity of the transcriptional start sites (TSSs; Fig. 3f,g). Consistently, 671 of 1,013 H3K9-increased regions (66.2%) were located within 500 bps of the TSSs of 633 genes (Supplementary Data 3). Similarly, 190 of 423 H4K16Ac-increased regions (44.9%) were located within 500 bps of the TSSs of 182 genes (Supplementary Data 4). Increased histone acetylation in the vicinity of TSSs suggests that expression of these genes may be increased^42^ in LTM maintenance. Besides increases in acetylation, we also detected their decreases: 1,042 regions with decreased H3K9Ac, 794 of which (76.2%) are within 500 bp of the TSSs of 744 genes (Supplementary Data 3) and 427 regions with decreased H4K16Ac, 366 of which (85.7%) are within 500 bp of the TSSs of 360 genes (Supplementary Data 4).

We also examined CRTC and CREB binding 1 day after spaced training. We identified 5,990 CRTC-binding sites, 4,995 of which (83.3%) were close to CREB-binding sites (Fig. 3h, Supplementary Fig. 6c,d and Supplementary Data 5). Although CREB showed few changes in binding after spaced training (239 out of 4,995 regions containing CREB/CRTC binding sites), CRTC binding was increased after spaced training (4,986 out of 4,995 regions containing CREB/CRTC-binding sites; Supplementary Data 5). The 4,986 region showing increased CRTC were mapped to 2,831 genes (Supplementary Data 5). Of the 2,831 CRTC-increased genes, 242 and 296 genes showed increases or decrease in H3K9Ac, respectively (Fig. 3i,j and Supplementary Data 3). Likewise, 102 and 80 genes showed increases or decreases in H4K16Ac, respectively (Fig. 3i,j and Supplementary Data 4). The overlaps between the gene lists showing increased CRTC and change in histone acetylation were significantly different from random (hypergeometric test in Fig. 3i,j), except for the overlap between the gene lists showing increased CRTC and decrease in H4K16Ac. We further performed Gene Ontology (GO) analysis using these overlapped gene lists and analysed clustering of GO-enriched terms. The clustered GO-enriched terms were mostly associated with neural functions (Supplementary Data 6 and the complete GO lists in Supplementary Data 7–9), although the clustered GO-enriched terms obtained from the gene lists showing increased CRTC and decreased H4K16Ac were related to the energy metabolism (Supplementary Data 6 and the complete GO list in Supplementary Data 10), which could contribute to the neural activities. Given the significant overlaps of CREB/CRTC binding and alteration in histone acetylation, CREB/CRTC in concert with histone acetylation could regulate the neural functions contributing LTM maintenance.

### Epigenetically modified target genes are required for LTM

**Figure 4:**
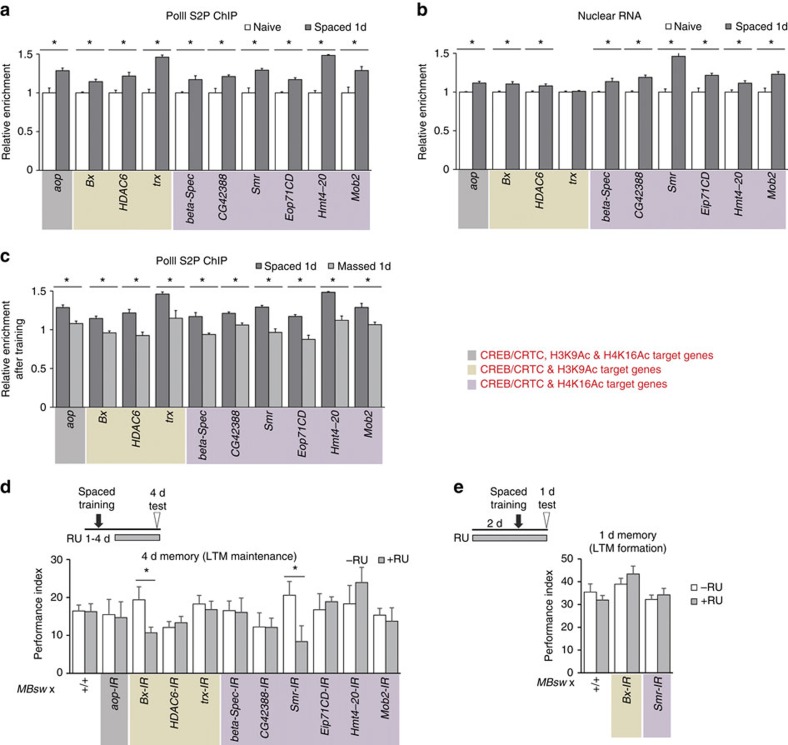
The CREB/CRTC and histone acetylation target genes required for LTM maintenance. (**a**) PolII S2P bindings to the candidate genes are increased 1 day after spaced training. The purified MB nuclei expressing FLAG-KASH prepared from naive control flies or flies at 1 day after spaced training were subjected to ChIP analysis using anti-PolII S2P (**a**) antibodies. The ChIP DNA was analysed at the indicated gene loci by qPCR (*n*=3 for all data). (**b**) Nuclear RNAs of the candidate genes are increased 1 day after spaced training. The MB nuclei were collected as in **a** and the nuclear RNAs were analysed by reverse transcriptase–qPCR (*n*=4 for all data). (**c**) PolII S2P bindings are abundant after spaced training, in comparison with massed training. The purified MB nuclei were subjected to PolII S2P ChIP analysis as in **a**. The naive control flies were used to calculate relative enrichment (*n*=3 for all data). (**d**) Knockdown of *Bx* and *Smr* impairs LTM maintenance (from left to right, *n*=10, 10, 8, 8, 8, 8, 8, 9, 9, 10, 8, 8, 12, 12, 8, 8, 8, 8, 8, 8, 8 and 8). (**e**) Knockdown of *Bx* and *Smr* does not affect LTM formation (from left to right, *n*=7, 8, 8, 8, 8 and 8). Wild-type flies (+/+) or the transgenic UAS-RNAi lines targeting the indicated genes were crossed with MBsw and their progeny were analysed as shown. Data from RU-fed flies (+RU) and the RU-unfed flies (−RU) were analysed. *P<0.05 determined by Student's *t*-test.

We next examined whether genes showing increased CREB/CRTC binding and H3K9 and/or H4K16 acetylation are required for 4-day LTM maintenance. Because of the large number of candidate genes, we selected ten genes (see Methods for selection of the genes), including histone modifiers from our candidate gene list (*HDAC6*, *trx* and *Hmt4-20*), to reveal the role of other histone modifications (Fig. 4).

We first confirmed that expression of our candidates was altered after LTM formation. Our method of purifying MB nuclei is not suited for measuring amounts of cytoplasmic messenger RNA transcripts; thus instead, we performed ChIP assays using antibody for Ser 2-phosphorylated RNA Pol II (PolII S2P), a marker for transcriptional elongation^43^,^44^. We found significant increases in PolII S2P binding at these genes 1 day after spaced training (Fig. 4a). To verify that increased PolII S2P binding correlates with increased transcription, we quantified nuclear RNA and found increases in expression of all candidate genes, except *trx*, 1 day after training (Fig. 4b). Increase in nuclear *trx* transcript might be transient and therefore it could be undetectable. PolII S2P binding was higher after spaced training compared with massed training (Fig. 4c), suggesting that increased expression of these genes is specific to LTM. These data are consistent with a model where CREB/CRTC activity and increased histone acetylation regulates expression of downstream genes, which could contribute to LTM maintenance.

We next used available RNAi lines to knock down expression of our candidate genes (Supplementary Fig. 2g–p), Of our ten candidate genes, knockdown of transcription factors, *Bx* and *Smr*, starting from 1 day after spaced training significantly impaired 4-day LTM (Fig. 4d). Knocking down these genes before training did not affect 1-day memory (Fig. 4e), indicating that they are required for 4-day LTM maintenance, but not formation. Similar to *CRTC*, expression of *Bx* and *Smr* in the α/βs neurons was required for 4-day LTM maintenance (Supplementary Fig. 7a–d).

**Figure 5:**
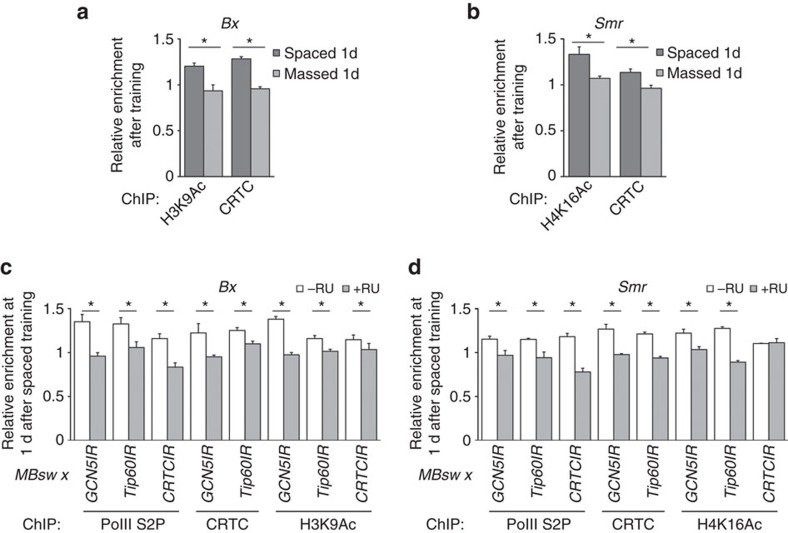
PolII S2P binding, CRTC binding and histone acetylation are regulated by GCN5, Tip60 and CRTC. (**a**,**b**) Histone acetylation and CRTC binding after spaced training are abundant in comparison with those after massed training at *Bx* and *Smr* gene loci. The purified MB nuclei expressing FLAG-KASH prepared from naive control flies or flies 1 day after spaced training or massed training were subjected to ChIP analysis using anti-H3K9Ac, anti-H4K16 or anti-CRTC antibodies. The ChIP DNA was analysed at *Bx* (**a**) or *Smr* (**b**). The naive control flies were used to calculate relative enrichment (*n*=3 for all data.). (**c**,**d**) PolII S2P binding, CRTC binding and histone acetylation are reduced by knockdown of *GCN5*, *Tip60* and *CRTC*. Flies expressing FLAG-KASH in the MBs and carrying MBsw were crossed with the UAS-RNAi lines, as indicated at the bottom of each panel. The MB nuclei from naive control flies and flies 1 day after spaced training were collected as in **a** and subjected to PolII S2P ChIP assay. The ChIP DNA was analysed at *Bx* (**c**) or *Smr* (**d**). The naive control flies were used to calculate relative enrichment (*n*=3 for all data). *P<0.05 determined by Student's *t*-test.

We then tested whether PolII S2P binding and CRTC binding at *Bx* and *Smr* gene loci were mediated by *GCN5*, *Tip60* or *CRTC* (Fig. 5). In addition, H3K9Ac at *Bx* and H4K16Ac at *Smr* were examined, which were determined by ChIP-seq analysis (Fig. 3e and Supplementary Fig. 6a). We first confirmed that those are specific to LTM protocol by ChIP–quantitative PCR (qPCR): H3K9Ac and CRTC binding at *Bx* or H4K16Ac and CRTC binding at *Smr* were significantly enriched after spaced training, in comparison with those after massed training (Fig. 5a,b). At the *Bx* gene locus, CRTC binding after spaced training depended on *GCN5* and *Tip60*, whereas PolII S2P and H3K9Ac after spaced training depended on expression of all three genes (Fig. 5c). This result is consistent with a model where GCN5 and Tip60 recruit CRTC to *Bx* and all three gene products together are required for acetylation of H3K9 and transcriptional activation. At *Smr*, binding of CRTC after spaced training also depends on *GCN5* and *Tip60*, and PolII S2P binding requires all three genes. However, acetylation of H4K16 occurs independently of CRTC (Fig. 5d). Collectively, these data suggest that 4-day LTM is achieved through GCN5 and Tip60, which increases CREB/CRTC-dependent transcription. Given that Bx and Smr are not required for 1-day LTM, but those expression is induced 1-day after spaced training, our data also suggest that ongoing transcription and the related memory performance are dissociated.

To determine whether *GCN5*, *Tip60* and *CRTC* contribute to basal expression of *Bx* and *Smr*, we measured bound PolII S2P in extracts from naive knockdown flies and found an effect when GCN5 was knocked down, but not Tip60 or CRTC (Supplementary Fig. 8a,b). Knocking down GCN5 did not affect H3K9Ac, suggesting that it may have a role in transcription, independent of its HAT activity (Supplementary Fig. 8a). Thus, in the naive state, basal expression of *Bx* and *Smr* may require GNC5 in a structural role^45^. Spaced training may function to both recruit Tip60 and CRTC, and also induce GCN5 HAT activity.

### Shifting transcriptional requirements for LTM maintenance

**Figure 6:**
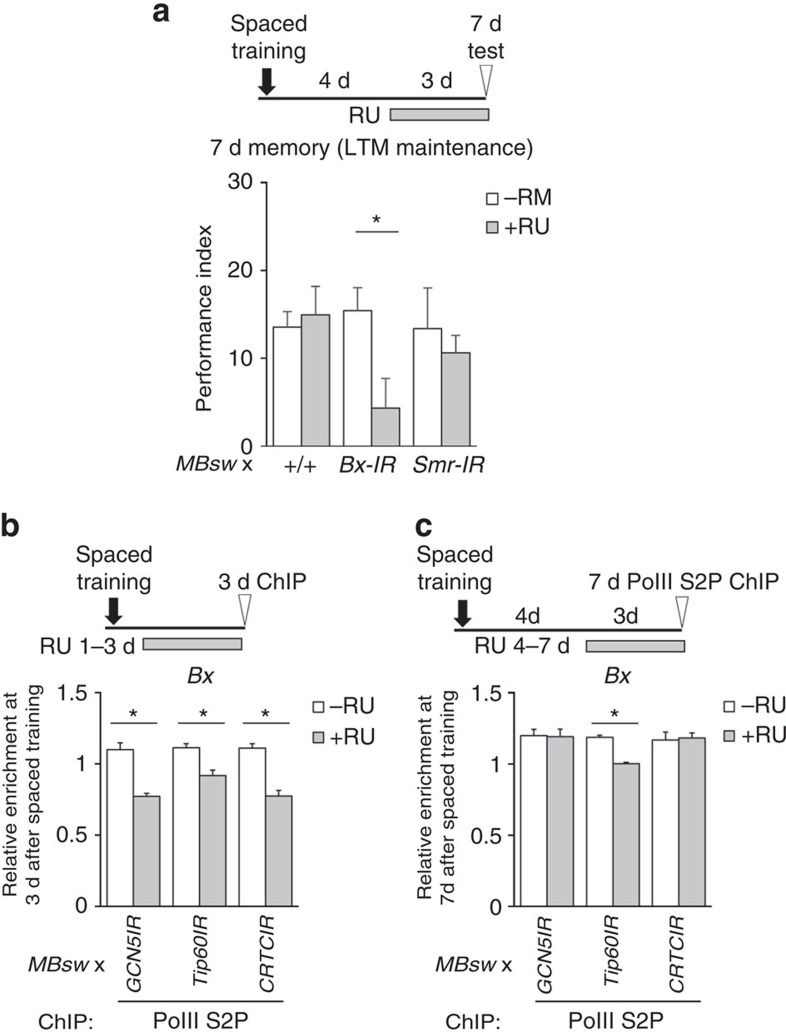
Shifting transcription system during LTM maintenance. (**a**) Knockdown of *Bx* impairs 7-day LTM maintenance. Wild-type flies (+/+) or the flies carrying the indicated UAS transgenes were crossed with MBsw and their progenies were tested as shown (from left to right, *n*=11, 10, 8, 8, 8 and 8). (**b**) Knockdown of *GCN5*, *Tip60* and *CRTC* impairs PolII S2P binding to *Bx* 3 days after spaced training. (**c**) Knockdown of *Tip60*, but not *CRTC* or *GCN5*, impairs PolII S2P binding to *Bx* 7 days after spaced training. Flies expressing FLAG-KASH in the MBs and carrying MBsw were crossed with the UAS-RNAi lines, as indicated at the bottom of each panel. Their progenies were subjected to ChIP assay, using the PolII S2P antibody. ChIP DNA was analyzed at *Bx*. Naive control flies with or without RU feeding and sampled at the same age after eclosion as the spaced trained flies were used to calculate relative enrichment after spaced training (*n*=3 for all data). *P<0.05 determined by Student's *t*-test.

Binding of PolII S2P at *Smr* and *Bx* remained high 3 days after training (Supplementary Fig. 9). This result is consistent with our observation that expression of *Smr* and *Bx* is required from 1 to 4 days after training for 4-day retention. However, we also found that PolII S2P binding continues to be elevated 7 days after training. This suggested to us that *Smr* and *Bx* may still be required after 4 days and we found that knockdown of *Bx*, but not *Smr*, from 4 to 7 days after spaced training impaired 7-day LTM (Fig. 6a).

Consistent with the requirement for CRTC, GCN5 and Tip60 in 4-day LTM maintenance, binding of PolII S2P to *Bx* 3 days after spaced training required *GCN5*, *Tip60* and *CRTC* (Fig. 6b). CRTC and GCN5 are no longer required for LTM maintenance 7 days after training (Figs 1d and 2c), suggesting that Bx expression may become CRTC and GCN5 independent 4 days after training. In agreement with this prediction, we found that PolII S2P binding to *Bx* 7 days after training still requires Tip60, but no longer requires CRTC or GCN5 (Fig. 6c). As *Bx* encodes a transcription factor, our data suggest the possibility that expression of maintenance genes may shift from being CREB dependent to Bx dependent.

### CREB/CRTC regulates LTM extinction

What is the biological meaning of the shift in transcription systems during LTM maintenance? Fasting activates CRTC by inducing nuclear translocation and this has been shown to accelerate LTM formation^24^. This raised the possibility that CRTC activity during early storage could function to strengthen LTM if additional training sessions are given at this time. To test this, we spaced trained flies and then trained them again 1 day later. Unfortunately, however, we did not observe any enhancement of LTM (Supplementary Fig. 10).

**Figure 7:**
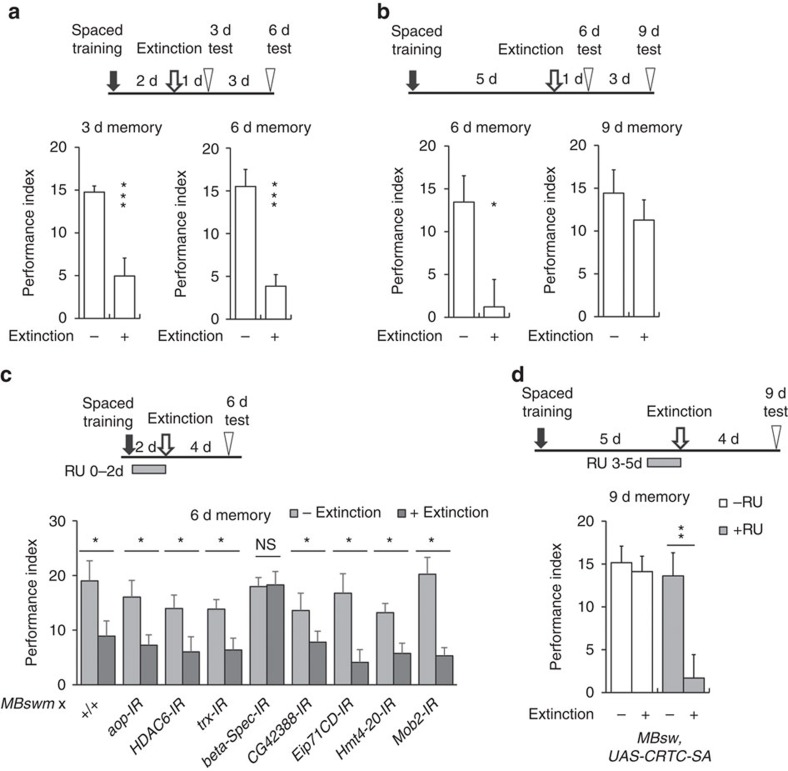
The CREB/CRTC target genes are required for LTM extinction. (**a**) Repeated exposure (15 times) to the learned odour (the extinction paradigm) at 2 days after spaced training results in suppression of 3-day LTM and 6-day LTM (from left to right, *n*=14, 12, 8 and 8). (**b**) The extinction paradigm at 5 days after spaced training results in suppression of 6-day LTM, but does not attenuate 9-day LTM (*n*=8 for all data). (**c**) Knockdown of *beta-Spec* impairs LTM extinction. Wild-type flies (+/+) or the UAS-RNAi lines targeting the indicated genes were crossed with MBsw and their progenies were analysed as shown. Data from flies that did not undergo the extinction paradigm (−Extinction) and flies subjected to the extinction paradigm (+ Extinction) were analysed by Student's *t*-test (from left to right, *n*=8, 8, 8, 8, 8, 8, 8, 8, 9, 10, 12, 12, 8, 8, 8, 8, 8 and 8). NS, not significant; *P<0.05, **P<0.01 and ***P<0.001.by Student's *t*-test. (**d**) CRTC-SA expression induces LTM extinction by the extinction paradigm at 5 days after spaced training. CRTC-SA was expressed by MBsw and tested as shown. (*n*=8 for all data; one-way analysis of variance; *P*=0.0011). **P<0.01 by Bonferroni testing.

In mice, it has been shown that CREB activity is required for memory extinction^46^. In addition, although recently formed memory is extinguishable, memory becomes resistant to behavioural extinction protocol over time^47^,^48^,^49^. Thus, we next tested whether CREB/CRTC-dependent transcription is associated with the ability to extinguish memories. We first examined whether LTM in *Drosophila* shifts from an extinguishable to an inextinguishable form over time. As previously reported^50^, when spaced trained flies were subjected to an extinction protocol (repeated exposures to the CS stimuli in the absence of the aversive unconditioned stimulus), 2 days after spaced training, they had attenuated LTM 1 day after extinction (Supplementary Fig. 11). LTM was still attenuated at 4 days after extinction (Fig. 7a). In contrast, when flies were subjected to extinction 5 days after spaced training, LTM was temporarily attenuated for 1 day, but recovered to normal within 4 days (Fig. 7b). These results suggest that persistent extinction of LTM only occurs within an early time window, when LTM is maintained by CREB/CRTC-dependent transcription system.

If extinction requires CREB/CRTC activity, CREB/CRTC target genes might consist of two classes: one class required for memory maintenance and a second class that allows extinction to occur on repeated unpaired CS exposure. Thus, we next screened our candidate genes using an extinction protocol and found that knockdown of *beta-Spec* completely suppressed extinction (Fig. 7c). *beta-Spec* was not involved in LTM maintenance (Fig. 4d) or formation (Supplementary Fig. 12), suggesting that it functions specifically to allow LTM to be modified or extinguished during CREB/CRTC-dependent maintenance.

If CREB/CRTC activity defines a time window for extinction, artificial activation of CRTC may prolong the time window for extinction. To test this idea, we expressed a constitutive active form of CRTC (CRTC-SA), which localizes to the nucleus in the absence of neuronal activity^24^,^51^, using the MBsw driver. When CRTC-SA was expressed in the MBs from 3 days to 5 days after spaced training, and flies were subjected to extinction 5 days after training, we observed attenuated LTM 4 days after extinction (Fig. 7d). Thus, the time window for LTM extinction can be extended by artificially manipulating CRTC activity.

## Discussion

**Figure 8:**
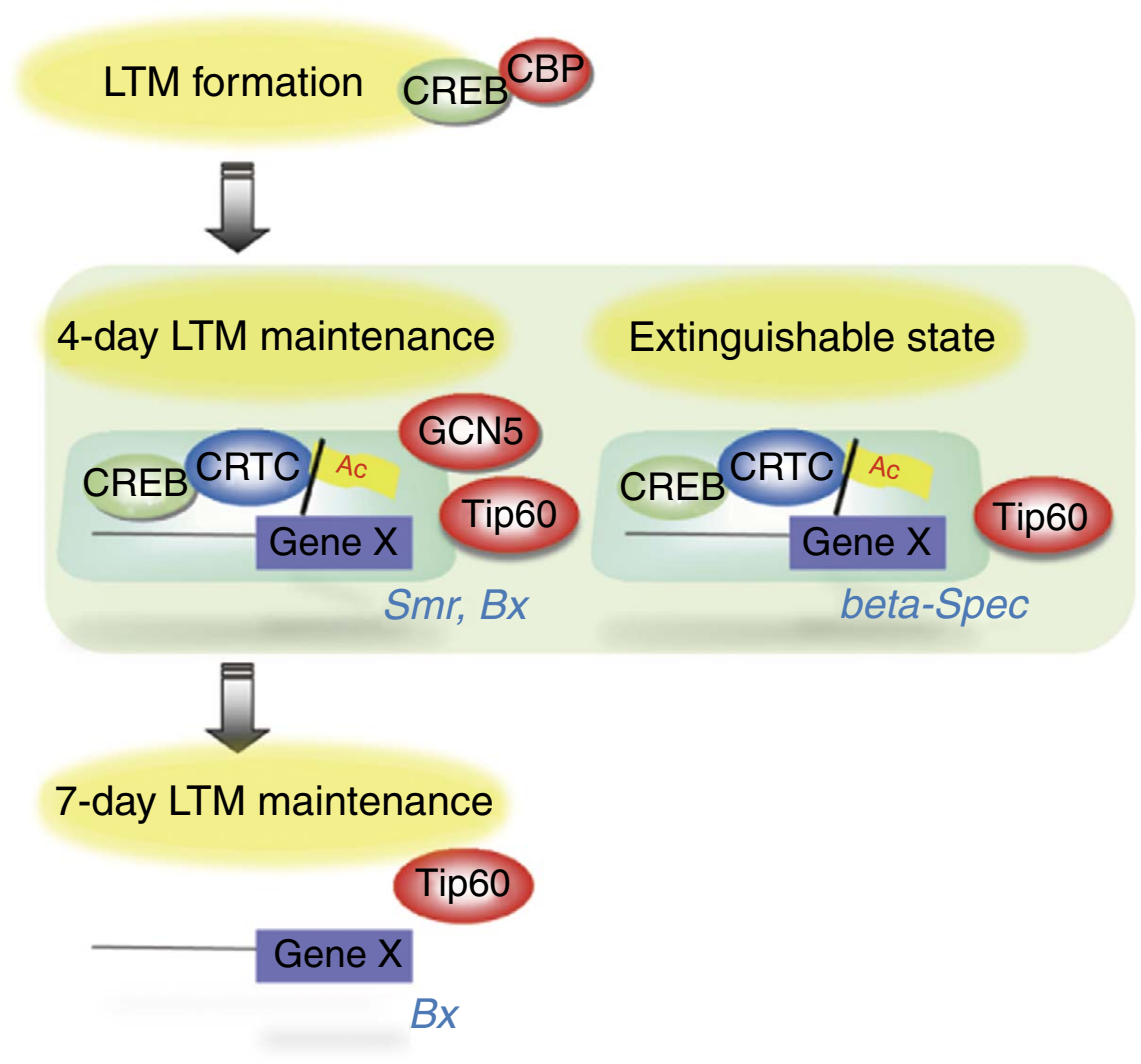
Shifting transcriptional system during LTM storage. Working model. LTM is formed through the activity of CREB/CBP. CREB/CRTC and HATs, GCN5 and Tip60 become essential for 4-day LTM maintenance by promoting expression of *Smr* and *Bx*. They also create an extinguishable state of LTM, by activating expression of *beta-Spec*. In later stage, activity of CREB/CRTC and GCN5 are no longer required; however, Tip60 and Bx are indispensable for 7-day LTM maintenance.

In this study, we demonstrate that maintenance of LTM requires activities of specific transcriptional systems distinct from those involved in formation. We further identify *Bx* and *Smr* as LTM maintenance genes and characterize a shift in transcription between CREB/CRTC-dependent maintenance (1–4 days) to Bx-dependent maintenance (4–7 days). In addition, we demonstrate a biological consequence of this shift in defining a time window during which LTM can be modified and identify a gene, *beta-Spec*, required for memory extinction (Fig. 8).

LTM maintenance mechanisms change dynamically during storage. In particular, CRTC, which is not required during memory formation, becomes necessary during 4-day LTM maintenance and then becomes dispensable again. Consistent with this, CRTC translocates from the cytoplasm to the nucleus of MB neurons during 4-day LTM maintenance and returns to the cytoplasm within 7 days. On the other hand, *Bx* expression is increased at both phases, suggesting that transcriptional regulation of memory maintenance genes may change between these two phases. Supporting this idea, we found that *Bx* expression requires CRTC during 4-day LTM maintenance but becomes to be independent of CRTC 7 days after training. We propose that CREB/CRTC activity induces *Bx* expression, which subsequently activates a feedback loop where Bx maintains its own expression and that of other memory maintenance genes.

Although we propose that the shifts in transcriptional regulation we observe occur temporally in the same cells, we cannot discount the possibility that LTM lasting 7 days is maintained in different cells from LTM lasting 4 days. MB Kenyon cells can be separated into different cell types, which exert differential effects on learning, short-term memory and LTM. Thus, it is possible that LTM itself consists of different types of memory that can be separated anatomically. In this case, CRTC in one cell type may exert non-direct effects on another cell type to activate downstream genes including *Bx* and *Smr*. However, as that CRTC binds to the *Bx* gene locus to promote Bx expression and both CRTC and Bx are required in the same α/βs subtype of Kenyon cells, it is likely to be that the shift from CRTC-dependent to Bx-dependent transcription occurs within the α/βs neurons.

Currently, we propose that the alterations in histone acetylation and transcription we uncovered are required for memory maintenance. However, we note that decreases in memory after formation could be caused by defects in retrieval and maintenance. Thus, it remains formally possible that the epigenetic and transcriptional changes we report here are required for recall, but not maintenance. We believe this is unlikely, as inhibition of CRTC from 4 to 7 days after memory formation does not affect 7 day memory, wherea inhibition from 1 to 4 days does. This suggests that at least one function of CRTC is to maintain memory for later recall.

Epigenetic regulations in neurons are required for LTM maintenance and LTM formation^14^,^16^,^52^,^53^. Consistent to the previous study in mice, which suggests distinct transcriptional regulations in LTM formation and maintenance^16^, our data indicate that memory formation and maintenance are distinct processes. Although the HAT, CBP, is required for formation but dispensable for maintenance, other HATs, GCN5 and Tip60, are required for maintenance but dispensable for formation. Through ChIP-seq analyses, we identified those downstream genes, *Smr* and *Bx*, as LTM maintenance genes and these are not required for LTM formation. Collectively, these results suggest differential requirements of histone modifications between LTM formation and maintenance. Although we identified other histone modifiers besides GCN5 and Tip60 in our screen, knockdown of these histone modifiers did not affect LTM maintenance. There are ∼50 histone modifiers encoded in the fly genome, raising the possibility that the lack of phenotype in some knockdown lines is due to compensation by other modifiers.

Our results indicate some correlation of increase in CRTC binding with histone acetylation and gene expression. Interestingly, DNA methylation shows higher correlation to gene expression in comparison with histone acetylation in mice^16^. Notably, flies lack several key DNA methylases and lack detectable DNA methylation patterns^54^. Hence, histone acetylation rather than DNA methylation may have a higher correlation with transcription in flies. We also detected reduction in histone acetylation overlapping with increase in CRTC binding. Those reductions could be due to CRTC interacting with a repressor isoform of CREB, CREB2b or other transcriptional repressor that binds near CREB/CRTC sites. These interactions would decrease histone acetylation and gene expression, and may be related to LTM maintenance. Although this study focused on the upregulation of gene expression through CREB/CRTC, downregulation of gene expression by transcriptional repressors may also be important in understanding the transcriptional regulation in LTM maintenance. Our results demonstrate the importance of HATs for LTM maintenance; however, our data do not conclude that histone acetylation is a determinant for gene expression, but rather it might be a passive mark of gene expression, as debated previously^7^,^8^. HATs also target non-histone proteins and also interact with various proteins, both of which could support gene expression in LTM maintenance^8^.

Similar to traumatic fear memory in rodents^47^,^48^,^49^, we found that aversive LTM in flies can be extinguished by exposing them to an extinction protocol specifically during 4-day LTM maintenance. These observations suggest the time-limited activation of molecules that allows LTM extinction only during the early storage. Supporting this concept, we found that CRTC is activated during the extinguishable phase of LTM maintenance and prolonging CRTC activity extends the time window for extinction. Thus, CRTC is the time-limited activated factor determining the time window for LTM extinction in flies. In cultured rodent hippocampal neurons, CRTC nuclear translocation is not sustained^26^, suggesting that other transcription factors may function in mammals to restrict LTM extinction.

Our work demonstrates that LTM formation and maintenance are distinct, and involve a shifting array of transcription factors, coactivators and HATs. A key factor in this shift is CRTC, which shows a sustained but time-limited translocation to the nucleus after spaced training. Thus, MB neurons recruit different transcriptional programmes that enable LTM to be formed, maintained and extinguished.

## Methods

### Fly stocks and culture conditions

The UAS-RNAi lines were obtained either from the Vienna *Drosophila* RNAi Center (Vienna, Austria) or National Institute of Genetics (Shizuoka, Japan) and *lexAop-rCD2::GFP* was a gift from M. Landgraf^55^. The split-GAL4 lines were a gift from G.M. Rubin^35^. The *UAS-CREB2-b* line was obtained from M. Ramaswami^56^, the *MBsw* (originally described as P{MB-Switch}12-1) lines from R. Davis^23^, the *UAS-CRTC-IR* line from M. Montminy^51^, the *MB247* line^57^ from T. Tabata, *UAS-nlsGFP* lines from Bloomington *Drosophila* Stock Center (Indiana) and the *hs-GAL4* line from the National Institute of Genetics. The *UAS-CRTC-HA* and the *UAS-CRTC-SA-HA* lines were obtained by germline transformation using standard procedures^24^. FLAG-KASH-expressing flies were obtained by germline transformation using standard procedures. Fly lines used in this study were outcrossed to our wild-type control line, w(CS10), for at least five generations before use, with the exception of the UAS-RNAi lines used in Fig. 4d. Flies were raised under a 12 h:12 h, light:dark cycle, at 23 °C and 60% humidity. All experiments were performed in light cycle. Both females and males were used for all experiments. Flies carrying MBsw were raised at 20 °C to reduce the possibility of leaky expression. RU (Mifepristone) was dissolved in ethanol and mixed with fly food, to a final concentration of 1% ethanol and 0.5 mM RU.

### Plasmid constructs

To construct the pMBpLexA-lexop-FLAG-KASH plasmid, a 10 × FLAG tag was fused to the carboxy-terminal domain of Klarsicht containing a KASH domain, corresponding to residues 1,860–2,262 (ref. 39), by PCR. The resulting 10 × FLAG-KASH fragment was cloned into pCasper-lexAop-W^55^ using the Gateway system (Life Technologies Corp., Carlsbad, CA, USA) according to the manufacturer's instructions. The MBpLexA fragment was cloned from pMBpLexA^40^ into plexop-FLAG-KASH, resulting in the pMBpLexA-lexop-FLAG-KASH construct.

### Single-cycle training and memory assays

Olfactory aversive conditioning was performed as previously described^58^,^59^. Briefly, ∼100 flies were placed in a training chamber, where they were exposed to odours and electrical shocks. In single-cycle training, one of the odours, either OCT (3-octanol) or MCH (4-methylcyclohexanol), was paired with electrical shocks (65 V, 1.5 s pulses every 5 s) for 1 min, whereas the other was not. For testing, flies were placed at a choice point between the two odours for 1.5 min. A performance index (PI) was calculated so that a 50:50 distribution (no memory) yielded a PI of 0 and a 0:100 distribution away from the shock-paired odour yielded a PI of 100. Individual performance indices were the average of two experiments, where the shock-paired odour was alternated.

### Spaced and massed training

Spaced and massed training were performed using an automated computer system, which controls both electric shock and odour application to flies. Spaced training consisted of five single-cycle training sessions (5 × spaced training), with a 15 min rest interval between each session^60^. Massed training was performed as five single-cycle training sessions without rest intervals. As previously described^17^, trained flies were rested at 17 °C. During RU feeding in the RNAi experiments, flies were transferred to 23 °C, to facilitate RU feeding. Memory was measured manually as described above.

### Extinction paradigm

The extinction paradigm was performed as previously described^50^, using the same automated computer system as spaced and massed training. The extinction paradigm consists of a repetition of single-cycle training sessions without rest intervals and electrical shocks. The spaced trained flies were rested at 17 °C for 1 day and transferred to 23 °C for recovery 1 day before the extinction paradigm. The flies were rested at 23 °C until testing.

### Quantification of transcripts (reverse transcriptase–qPCR)

Total RNA from *Drosophila* heads was extracted with TRIzol reagent (Thermo Fisher Scientific, San Jose, CA, USA) and complementary DNA was synthesized using ReverTra Ace qPCR RT Master Mix with gDNA Remover (TOYOBO, Osaka, Japan). cDNAs were then analysed by quantitative real-time PCR (BioRad Laboratories, Hercules, CA, USA). Transcripts of *rp49* were used for normalization.

### Purification of MB nuclei

Approximately 500 and 1,000 flies expressing FLAG-KASH in the MBs were used for ChIP and for ChIP-seq analysis, respectively. Heads were collected and homogenized in crosslinking buffer (15 mM Hepes-KOH at pH 7.5, 100 mM NaCl, 0.1% NP40, 1 mM dithiothreitol and 1 mM phenylmethylsulfonyl fluoride) containing 1% formaldehyde, Complete Protease Inhibitor Cocktail, PhosSTOP (Roche Diagnostics, Indianapolis, IN, USA) and 5 mM sodium butylate, using a Teflon/glass homogenizer. The homogenate was left on ice for 15 min. Crosslinking was quenched by adding 125 mM glycine and the homogenate was filtered through 40 μm nylon mesh to remove cuticles. The nuclei were rinsed three times in extraction buffer (20 mM Tris-HCl at pH 8.0, 100 mM NaCl, 1 mM EDTA, 1% Triton X-100 and 0.1% SDS). The nuclei were dissolved in extraction buffer, containing 1% BSA, Complete Protease Inhibitor Cocktail, PhosSTOP (Roche Diagnostics) and 5 mM sodium butylate, briefly sonicated to dissociate the individual nuclei, and precipitated with ANTI-FLAG M2 Affinity Gel (Sigma, St Louis, MO, USA). The precipitates were rinsed four times in extraction buffer, with 5 min nutation at 4 °C between washes and subjected to ChIP analysis.

Nuclei were stained with propidium iodide (Sigma; Fig. 3b,c). To stain purified MB nuclei with anti-FLAG antibody (Fig. 3d), precipitated MB nuclei were released from the beads by incubating in extraction buffer containing 100 μg ml^−1^ 3 × FLAG Peptide (Sigma). The released nuclei were rinsed to remove 3 × FLAG Peptide, stained with mouse anti-FLAG antibody (Sigma) at 1,000-fold dilution, followed by incubation with donkey anti-mouse IgG Alexa Fluor 488 antibody (Invitrogen, CA, USA) at 500-fold dilution.

To quantify the purity of the MB nuclei, the DNA was extracted from the precipitates once with phenol:chloroform and once with chloroform, and then ethanol precipitated. The resulting DNA (ChIP DNA) was eluted in TE buffer and quantified by quantitative real-time PCR (Supplementary Fig. 4b). Although this properly quantifies the amount of immunoprecipitated nuclei, we could have underestimated the purity. We have assumed the same amount of the background precipitates in the tagged and untagged samples; however, the FLAG-KASH-tagged nuclei could mask the beads and lower the background.

### ChIP analysis

The ChIP analysis was performed as previously described^61^, with modifications. The purified MB nuclei dissolved in extraction buffer containing 0.1% BSA, Complete Protease Inhibitor Cocktail, PhosSTOP (Roche Diagnostics) and 5 mM sodium butylate were sonicated using a Q500 Sonicator (QSonica, Newtown, CT, USA), at a power setting of 20%, for a total of 3 min, resulting in fragmentation of DNA ranging from 100 to 500 bp. The extracts were centrifuged to remove insoluble materials including the M2 Affinity Gel and the supernatants were used for immunoprecipitation with 10 μl of Protein A/G Agarose (Thermo Fisher Scientific) and the following antibodies: 4 μg of anti-H3K9Ac (ab4441, Abcam, Cambridge, MA, USA), 4 μg of anti-H4K16Ac (07–329, Merck Millipore, Bedford, MA, USA), 1 μg of anti-PolII S2P (ab5095, Abcam), 4 μg of anti-CREB and 4 μg of anti-CRTC antibodies. After overnight incubation at 4 °C, the beads were washed four times with high-salt buffer (20 mM Tris-HCl at pH 8.0, 500 mM NaCl, 1 mM EDTA, 1% Triton X-100 and 0.1% SDS), with 5 min nutation at 4 °C between washes. The beads were rinsed with 1 × TE buffer (10 mM Tris-HCl at pH 8.0 and 1 mM EDTA) and the immunoprecipitates were eluted with 1 × TE buffer containing 1% SDS. Crosslinking was reversed by incubation at 65 °C for 6 h. Next, samples were incubated with Proteinase K (Takara, Shiga, Japan) at 55 °C for 1 h, the DNA was extracted once with phenol:chloroform and once with chloroform, and then ethanol precipitated. The resulting DNA (ChIP DNA) was eluted in TE buffer containing 100 μg ml^−1^ transfer RNA and quantified by quantitative real-time PCR, using primer pairs amplifying the peak regions of H3K9Ac, H4K16Ac or CRTC (Supplementary Data 11). The primer pairs used in ChIP analysis for PolII S2P amplify a region 0.5–1 kb downstream of TSS, which indicates transcriptional elongation^43^. To obtain relative enrichment at each gene locus, the amount of DNA at each locus was normalized by the internal control, *elav*, a neuronal marker gene, except for ChIP analysis of CRTC. In CRTC-ChIP analysis, the amount of precipitated DNA from each gene locus was normalized by the input DNA. Normalization using internal controls, such as *elav*, gives more stable results compared with normalization using input DNA, as it excludes the variation in DNA loss during sample preparation. However, CRTC binding increased at all CRTC-binding sites, including *elav*, so that an internal control was not used for normalization in CRTC-ChIP analysis.

### ChIP-seq analysis and bioinformatic analysis

The ChIP DNA samples prepared as described above were used to prepare a library for SOLiD5500 (Thermo Fisher Scientific) to detect histone acetylation or MiSeq (Illumina, San Diego, CA) to detect CREB and CRTC binding, according to the manufacturer's instructions. Single- and paired-end reads were generated for ChIP and input DNA in SOLiD5500 and Miseq, respectively. The data were processed as follows.

The reads obtained using Miseq for CREB/CRTC ChIP-seq read quality was first assessed using FastQC (version 0.11.4) (http://www.bioinformatics.babraham.ac.uk/projects/fastqc/), to identify low average quality, adaptor contamination or PCR duplications (Supplementary Data 1). The results were shown as ‘PASS', ‘WARN' (warning) or ‘FAIL' (failure) based on its criteria (Supplementary Data 1). Probably because the ChIP libraries are inherently biased in their sequence composition, some test elicited the warning or failure. Nonetheless, those did not affect the mapping efficiency (Supplementary Data 2). In the case of the reads obtained by SOLiD5500 for histone acetylation ChIP-seq, due to the colourspace coding in the SOLiD system, which estimates quality value based on the ‘colour quality', the original raw reads did not pass ‘per base sequence quality'. After the alignment, the base quality of the mapped reads was recalculated from the quality value by Bioscope, which passed the base quality criteria in FastQC (Supplementary Data 1).

CLC Genomics Workbench (CLC Bio, Aarhus, Denmark) was used to filter the reads obtained using Miseq at the default parameter of (cutoff less than the quality value 14 and length cutoff <16 bp) and map the filtered reads to the *Drosophila* reference genome, dm3 (dmel_r5.43) from the University of California, Santa Cruz (UCSC). The paired reads 500 bp apart from each other were eliminated. LifeScope Genomic Analysis Software (Thermo Fisher Scientific) was used to map the raw reads obtained by SOLiD5500 to dm3 (dmel_r5.43). In both cases, the reads with low mapping quality below 8 were eliminated and the retained reads (Supplementary Data 2) were analysed as follows.

The sequence depth was assessed to confirm that sequencing was deep enough to reproduce the data, using the saturation correlation in the MEDIPS R package^62^. Briefly, the mapped reads in one sample were split into two, to see whether the split reads correlates each other. The estimated correlation was >0.85 in all samples, except input samples (0.64 and 0.76), suggesting that all ChIP samples were sequenced deep enough to reproduce data.

The correlation between three biological replicates were evaluated by the Pearson's correlation in the MEDIPS R package. The Pearson's correlation was >0.9 in any pair of biological replicates. In addition, the principal component analysis was performed using the Bioconductor package DESeq2 (ref. 63 and Supplementary Fig. 5), with the windowed data set described below, indicating that the distribution of the reads in each samples clusters into their respective groups. Aggregate gene plot was shown by ngs.plot^64^ using the mapped reads in which the reads from three biological replicates were pooled.

The peaks were called by comparing ChIP DNA with input DNA, using PICS^65^,^66^ and ERD algorithms on Strand NGS software (Agilent Technologies, Palo Alto, CA, USA). The overlapping regions of two criteria were determined as peaks. The PICS and ERD were run using a default settings, except for the following parameters: for PICS, 120 bp as an average fragment length, 10 bp as a minimum distance between forward and reverse reads, 200 bp as a maximum distance between forward and reverse reads, 100 bp as a window width, with 5% false discovery rate; for ERD, 2 as an enrichment factor, 100 bp as a window size, 10 bp as a window slide size and 100 bp as a minimum region size. The peaks obtained by ERD analysis were filtered by an enrichment factor of 2 and a density of reads at 0.3 for H3K9Ac, 0.15 for H4K16Ac, 0.12 for CREB and 0.2 for CRTC. In CREB and CRTC ChIP-seq, the peaks called at least two out of three biological replicates were determined as the CREB- or CRTC-binding sites. Those located within 200 bp were defined as CREB/CRTC-binding sites (Supplementary Data 5).

In the analysis of histone acetylation, we first pooled all peaks called in three replicates excluding the overlapping peaks and then created the 400 bp windows centring the called peaks. The overlapping regions in the adjacent windows were trimmed, and if the trimmed windows were <200 bp the contiguous windows were merged. The reads were counted in the individual windows and analysed by the Bioconductor package DESeq2 (ref. 63). An adjusted *P*-value<0.1 was considered to be statistically significant (Supplementary Data 3 and 4).

To analyse CREB and CRTC binding, the 1 kb windows covering the entire genome were created and the mapped reads in each windows were counted. The CRTC binding was increased at almost all binding regions. When the reads were counted only from the peak regions, the normalization step in DESeq2 equalizes the read counts, thereby resulting in no significant increase. However, by including the background reads outside of the peaks using the 1 kb window setting, analysis in DESeq2 showed significant increase in CRTC binding (Supplementary Data 5).

The hypergeometric test, phyper in R, was applied to test the overlapping gene lists in Fig. 3i,j.

GO-enriched groups and the following functional clustering were determined by GO analyses using the Database for Annotation, Visualization and Integrated Discovery^67^. Briefly, the input genes, such as CREB/CRTC target genes, were functionally annotated by GO terms and the number of the genes in each GO terms were counted. These counts were compared with the reference counts in each GO terms obtained from the total genes in the genome, by a modified Fisher's exact test, which identifies GO -enriched group of the given gene list.

From the initial pilot experiment without biological replicates, a total of 50 candidate genes showing increase in histone acetylation and CREB/CRTC binding were obtained. To confirm that we focused on positive hits, almost all candidate's RNAi were behavioural examined, whereas the biological replicates of ChIP-seq were analysed. After the multiple comparisons and proper false discovery rate estimations, the candidate genes were downscaled to ten genes.

### Quantification of nuclear RNAs

The MB nuclei were prepared as in ChIP assay, using ∼500 flies expressing FLAG-KASH in the MBs, with Ribonuclease Inhibitor at 60 U ml^−1^ (Takara). The MB nuclei collected by immunoprecipitation were dissolved in 1 × TE buffer containing 1% SDS with Proteinase K (Takara) and incubated at 65 °C for 6 h to recover crosslinking. RNA was extracted once with phenol:chloroform and once with chloroform, and then ethanol precipitated. The resulting RNA was used to synthesize cDNA by ReverTra Ace qPCR RT Master Mix with gDNA Remover (TOYOBO). cDNAs were then analysed by quantitative real-time PCR (BioRad Laboratories). Transcripts of *rp49* were used for normalization.

### Generation of antibodies

Anti-CREB antibody was raised against full-length CREB2b, which covers all major isoforms^68^, and the anti-CRTC antibody was raised against the C-terminal region of CRTC, corresponding to residues 330–789, by Japan lamb (Hukuyama, Hiroshima, Japan). Sera were collected and purified using a resin conjugated with the antigens.

### Immunohistochemistry

Flies were anaesthetized on ice, their proboscides were removed and then they were fixed in 4% paraformaldehyde in PBS for 30 min on ice. Fixed brains were dissected and subjected to whole-mount immunohistochemistry. Brains were treated with PBS containing 0.3% Triton X-100 for 1 h, blocked for 1 h with PBS containing 0.1% Triton X-100 and 4% Block Ace (Sumitomo Dainippon Pharma, Osaka, Japan) and incubated with antibodies diluted in blocking solution for 1 day at 4 °C. Antibodies used were mouse anti-FLAG antibody (Sigma) at a 1,000-fold dilution, mouse anti-HA antibody (Covance, NJ, USA) at a 50-fold dilution, mouse anti-nc82 (Developmental Studies Hybridoma Bank, University of Iowa, USA) detecting a presynaptic protein, Bruchpilot, at a 50-fold dilution and chicken anti-GFP antibody (ab13970, Abcam) at a 2,000-fold dilution. Brains were then incubated with donkey anti-mouse IgG Alexa Fluor 488 antibody (Thermo Fisher Scientific), donkey anti-chicken IgY Alexa 488 antibody (Jackson ImmunoResearch Labs, Inc., West Grove, PA, USA) and goat anti-mouse IgG Alexa Fluor 555 antibody (Thermo Fisher Scientific) diluted 1:250 in blocking solution, for 6 h at room temperature. Nuclei were stained with TO-PRO-3 iodide, diluted 1:500 in PBST, for 30 min at room temperature. Brains were mounted in PermaFluor (Lab Vision Corp., Fremont, CA, USA) and images were captured using a confocal microscope. The confocal images were processed using the deconvolution software, Autoquant X3.

### Quantification of nuclear CRTC accumulation

Quantification of nuclear CRTC-HA was performed as previously described^24^. Data acquired at the same time under identical microscope settings were analysed as follows. One or two optical slices were selected, containing ∼100–200 nuclei and the dendritic region, the calyx. Neurons expressing CRTC-HA were clipped. CRTC-HA signal intensities overlapping with nuclear staining were normalized to those in the calyx. The median nuclear CRTC-HA signal intensities in each brain were then averaged. To evaluate the population of the MB neurons showing nuclear accumulation of CRTC, the number of the nuclei showing CRTC-HA signal intensities more than that in calyx were counted.

### Statistics

No statistical methods were used to predetermine sample sizes, but our sample sizes are similar to those reported in previous publications^17^,^22^,^23^,^24^,^31^,^50^. Statistical analyses were performed using Prism version 5.0. Two-tailed unpaired Student's *t* test was used for comparisons between two groups. The simultaneously tested groups were statistically compared, as absolute performance indices are not always stable when data were taken on different days, due to experimental conditions. When multiple groups were analysed, one-way analysis of variance followed by Bonferroni *post-hoc* comparisons were used for comparisons of multiple groups. Median CRTC signal intensities in nuclei and median number of nuclei showing nuclear CRTC were analysed by the Mann–Whitney *U*-test or by a two-way analysis of variance, followed by Bonferroni testing in comparisons between two groups, and in comparisons of multiple groups, those were analysed by the Kruskal–Wallis test followed by Dunn's multiple comparisons test. *P*-values<0.05 were judged to be statistically significant. All data in bar graphs were presented as a mean±s.e.m. The initial placement of the flies in different conditions, such as RU-fed or RU-unfed, was randomized. No data points were excluded. Data collection and analyses were not performed blind to the conditions of the experiments. The variance was tested in each group of the data and the variance was similar among genotypes. Data distribution was assumed to be normal, but this was not formally tested. The data were collected and processed randomly. Each experiment has been successfully reproduced at least two times and was performed on multiple days.

### Data availability

The data sets generated during and/or analysed during the current study are available from the corresponding author upon request.

The GEO accession numbers for the ChIP-seq data reported in this study are GSE73386 for histone acetylation and GSE81456 for CREB/CRTC.

## Additional information

**How to cite this article:** Hirano, Y. *et al.* Shifting transcriptional machinery is required for long-term memory maintenance and modification in *Drosophila* mushroom bodies. *Nat. Commun.* **7,** 13471 doi: 10.1038/ncomms13471 (2016).

**Publisher's note:** Springer Nature remains neutral with regard to jurisdictional claims in published maps and institutional affiliations.

## Supplementary Material

Supplementary Information Supplementary Figures 1-12

Supplementary Data 1 FastQC result of the sequencing reads

Supplementary Data 2 Alignment summary in ChIP-seq

Supplementary Data 3 H3K9Ac-altered genes

Supplementary Data 4 H4K16Ac-altered genes

Supplementary Data 5 CREB/CRTC-bound genes

Supplementary Data 6 Clustering the GO-tems

Supplementary Data 7 GO analysis of the CREB/CRTC target genes with increased H3K9Ac

Supplementary Data 8 GO analysis of the CREB/CRTC target genes with decreased H3K9Ac

Supplementary Data 9 GO analysis of the CREB/CRTC target genes with increased H4K16Ac

Supplementary Data 10 GO analysis of the CREB/CRTC target genes with decreased H4K16Ac

Supplementary Data 11 Primers used in this study

Peer Review File
