# Supplementary material for: Shifting transcriptional machinery is required for long-term memory maintenance and modification in Drosophila mushroom bodies
Source: Nat Commun. 2016 Nov 14;7:13471. doi: 10.1038/ncomms13471 (PMC5114576; doi:10.1038/ncomms13471)
Supplement: Supplementary Information — Supplementary Figures 1-12 [file ncomms13471-s1.pdf]

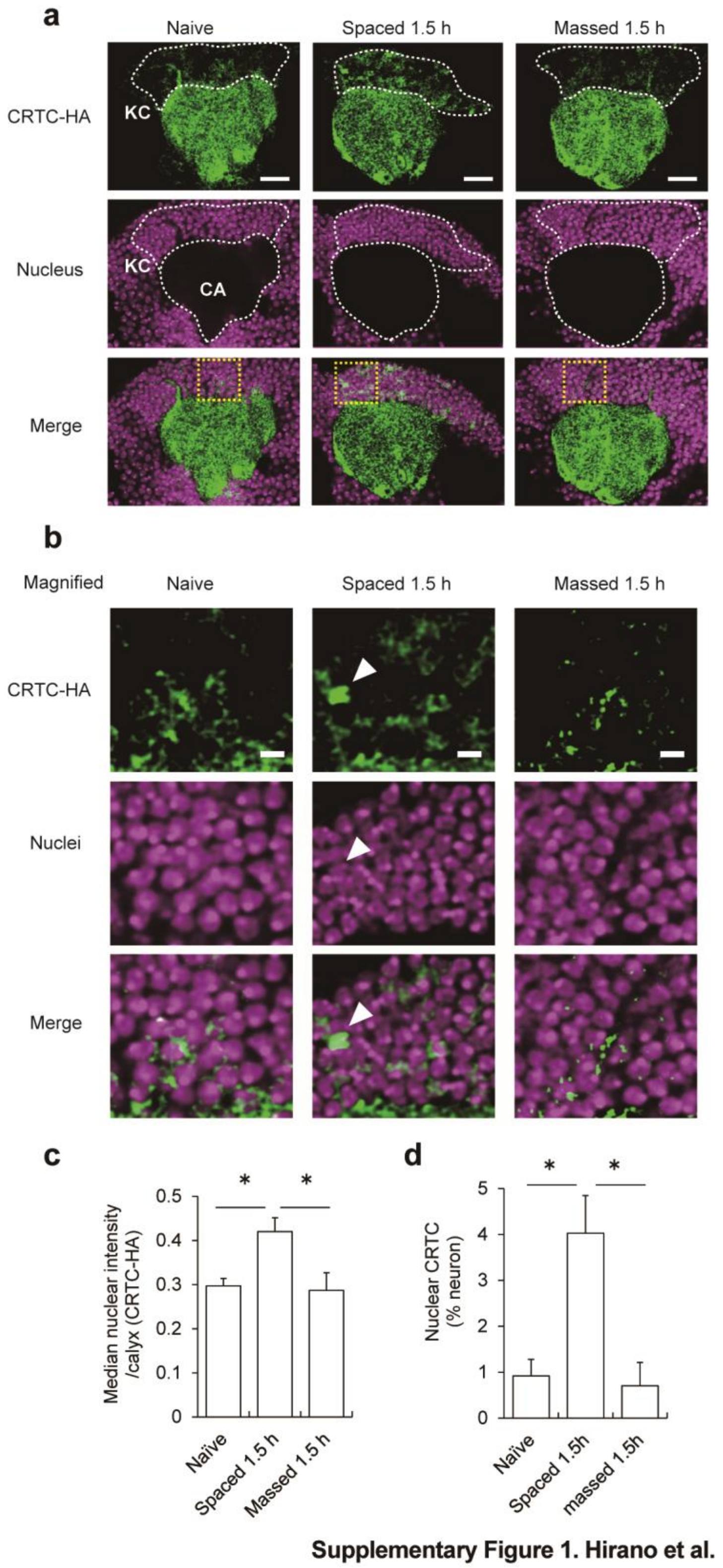

Supplementary Figure 1 Spaced training induces nuclear accumulation of CRTC in the MBs.

(a) Subcellular localization of CRTC in the MBs 1.5 hours after spaced training. *UAS-CRTC-HA* flies were crossed with a MB GAL driver, *MB247*. CRTC-HA was stained using anti-HA antibodies (green) and the nuclei were stained by TO-PRO-3 iodide (magenta). The MB cell bodies, Kenyon Cells (KC), and the dendritic region, calyx (CA) are indicated by white dotted lines. Scale bars, 10 μm.

(b) Magnified view of the yellow squared area in (a). White arrow heads indicates nuclear translocation of CRTC. Scale bars, 2 μm. The images are representative of 4-5 experimental replicates which are quantified in **c** and **d**.

(c) Median nuclear CRTC-HA intensity is increased 1.5 hours after spaced training. The individual nuclear CRTC-HA signal intensities were normalized to those in the calyx. The medians of nuclear CRTC-HA signal intensities in each brain were obtained, and the averaged medians were shown (from left to right, n =5, 4 and 4). Data were analyzed by Kruskal-Wallis test ($p = 0.0047$) followed by Dunn's Multiple Comparisons Test (*, $p < 0.05$).

(d) Midian number of the nuclei containing more CRTC-HA intensities than the calyx (from left to right, n =5, 4 and 4). Data were analyzed by Kruskal-Wallis test ($p = 0.0109$) followed by Dunn's Multiple Comparisons Test (*, $p < 0.05$).

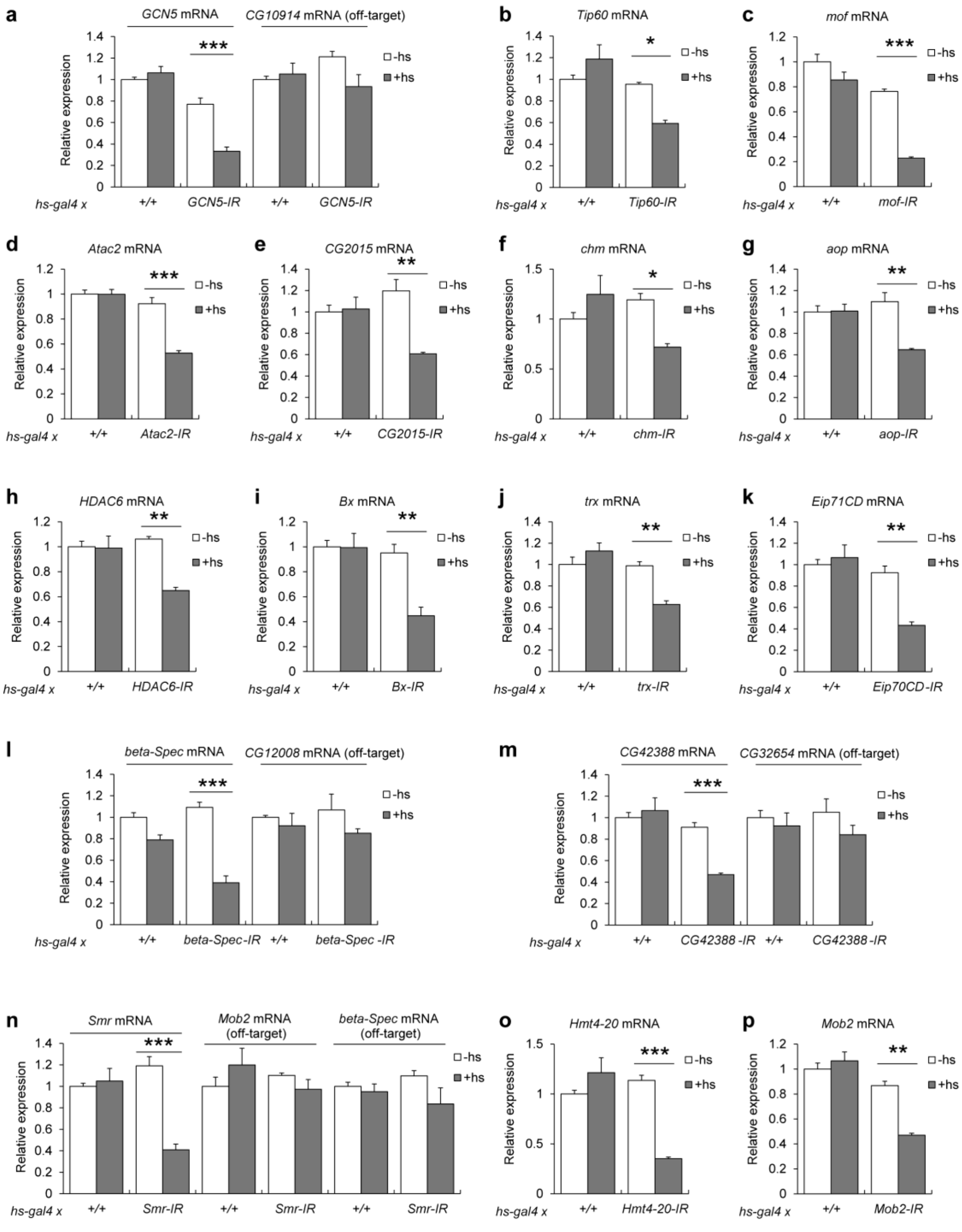

Supplementary Figure 2. Hirano et al.

Supplementary Figure 2 Knockdown efficacy by expressing the inverted repeats (IR).

Wild-type flies (+/+) or the transgenic flies carrying each *UAS-IR*s indicated at the bottom were crossed with *heat-shock GAL4* driver (*hs-gal4*), and their progenies were raised at 17°C. After eclosion, the flies were heat-shocked at 37°C for 30 min and rested at 23°C. The flies were

similarly heat-shocked the next day, and then collected at the following day (+hs). The flies without heat-shock were kept at 17°C (-hs). RNAs extracted from fly heads were analyzed by RT-qPCR. The sequences of *GCN5-IR*, *beta-Spec-IR, CG42388-IR*, and *Smr-IR* were predicted to have off-targets (possible knockdown of *CG10914* by *GCN5-IR*, *CG12088* by *beta-Spec-IR*, *CG32654* by *CG42388-IR*, and *Mob2* and *beta-Spec* by *Smr-IR*), based on 19-mer or more perfect matches in the RNAi sequence to other gene (Kulkarni M., et al, 2006, Nature Method, vol3, p833-838). The 19-mer perfect matches were screened using dscheck (http://dscheck.rnai.jp/). The expression of the off-target genes was not altered. The ratio to *rp49* expression was analyzed by One-way ANOVA followed by Bonferroni testing. *, $p < 0.05$, **, $p < 0.01$, ***, $p < 0.001$. n = 4 for all data .

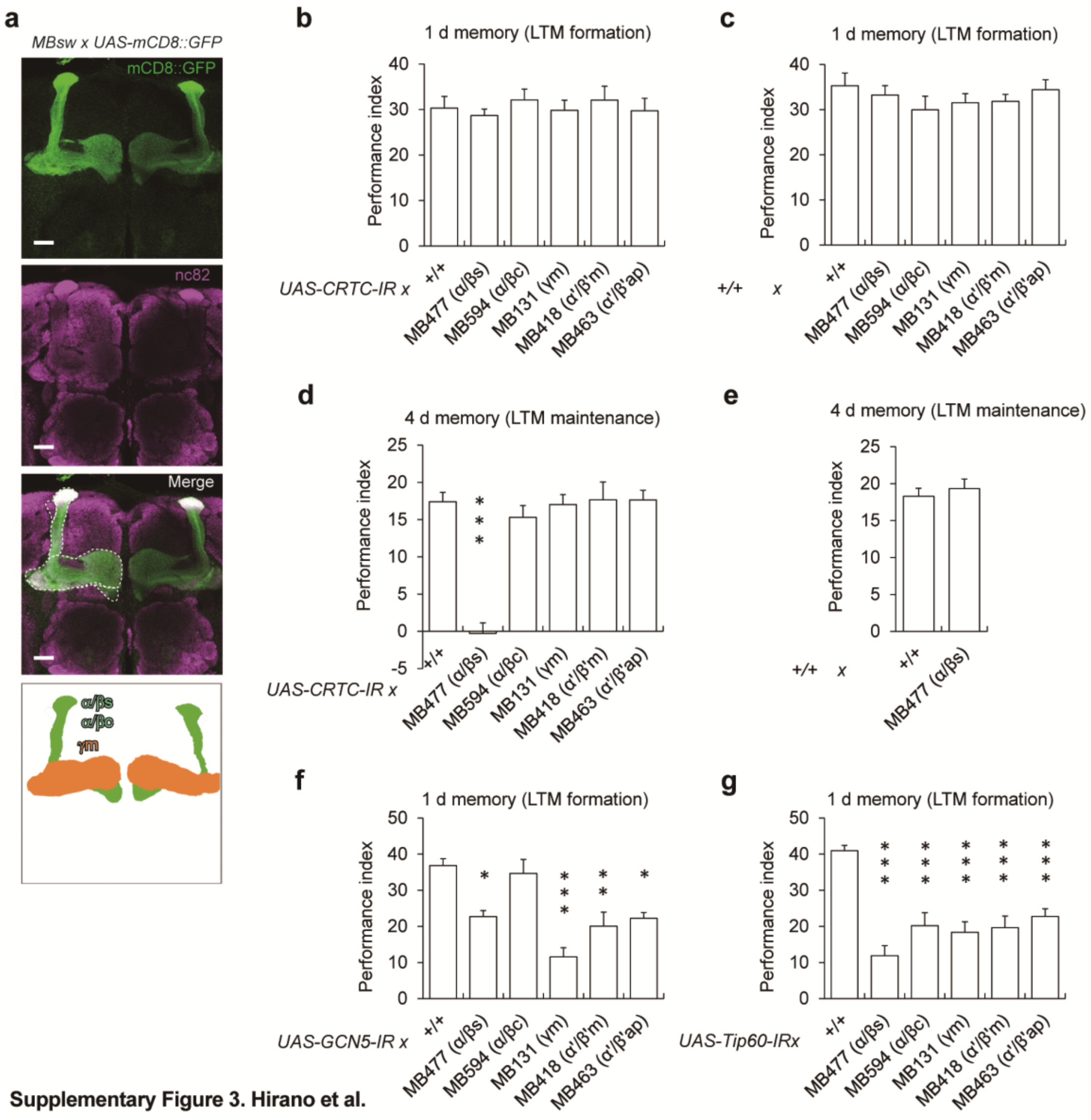

Supplementary Figure 3 Knockdown of *CRTC* in the α/βs neurons impairs 4-day LTM maintenance, and knockdown of *GCN5*, *Tip60* and *CBP* results in developmental defects.

(a) The MB lobes labelled by MBsw. MBsw flies were crossed with *UAS-mCD8::GFP* flies. The progeny was fed RU for 2 days and analyzed using a confocal microscope. GFP was visualized using anti-GFP antibodies (green), and presynaptic sites were visualized by the nc82 antibodies (magenta). The projection of a confocal stack is shown. All MB neurons were delineated by white dotted lines. MBsw labels the α/β and γ neurons, which contains the α/β

surface (α/βs), the α/β core (α/βc) and γ main (γm) neurons. Scale bars represent 20 μm. The images are representative of 3 experimental replicates.

(b, c) Knockdown of *CRTC* in the MB subpopulations doesn't affect LTM formation (**b**, from left to right, n = 8, 12, 8, 11, 8 and 8; **c**, n = 8 for all data).

(d, e) Knockdown of *CRTC* in the α/βs neurons impairs 4-day LTM maintenance (**d**, from left to right, n = 8, 12, 12, 8, 12 and 8; **e**, n = 8 and 8).

(f) Knockdown of *GCN5* in the α/βs, γm, α'/β'm and α'/β'ap neurons impairs LTM formation (from left to right, n = 8, 8, 8, 8, 12 and 8).

(g) Knockdown of *Tip60* in the α/βs, α/βc, γm, α'/β'm and α'/β'ap neurons impairs LTM formation (from left to right, n = 10, 8, 8, 8, 12 and 8).

Wild-type flies (+/+) or the transgenic flies carrying each *UAS-IRs*.were crossed with the split-GAL4 driver lines, named as MB series indicated at the bottom. The neuropils labeled by each split-GAL4 drivers were indicated in parentheses. 1-day memory (LTM formation) or 4-day memory (4-day LTM maintenance) after spaced training were tested.

Data was analyzed by One-way ANOVA followed by Bonferroni testing. *, $p < 0.05$, **, $p < 0.01$, ***, $p < 0.001$.

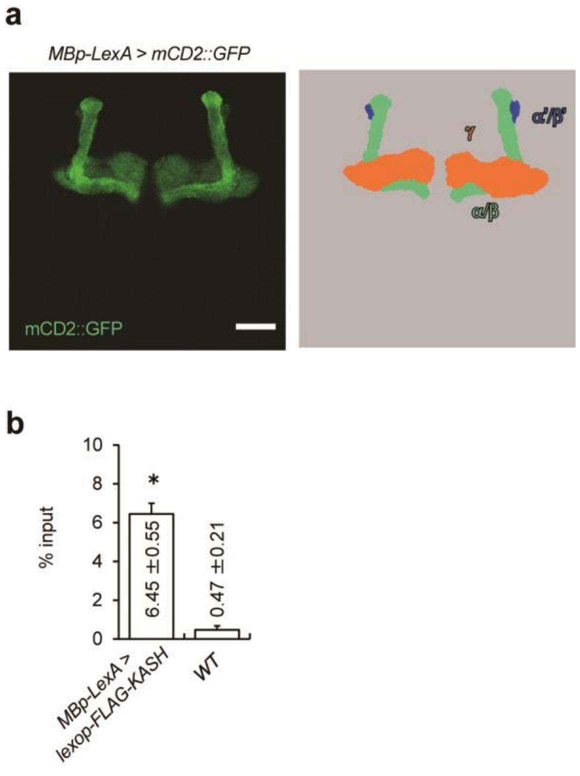

Supplementary Figure 4. Hirano et al.

Supplementary Figure 4 Purification of the MB nuclei using *MBp-LexA FLAG-KASH* flies.

(a) The MB lobes labelled by *MBpLexA*. *MBpLexA* flies were crossed with *lexAop-rCD2::GFP* flies, and the progeny was analyzed using a confocal microscope. GFP was visualized using anti-GFP antibodies (green). The projection of a confocal stack is shown. Scale bars represent 50 μm. The images are representative of 2 experimental replicates.

(b) The efficiency of the isolation of nuclei expressing FLAG-KASH. Nuclei prepared from the heads of the *MBpLexA, lexop-FLAG-KASH* flies or wild-type (WT) flies were immunoprecipitated with anti-FLAG antibodies. DNA was recovered from precipitated and input nuclei. Relative DNA amounts from precipitates compared to inputs were determined by quantitative real-time PCR, using a primer set to amplify *gapdh2* (n=3 for both data). Given that the background precipitates from WT were 0.47% of input, the purity of MB nuclei, 5.98% out of 6.45%, was 92.7%. p, < 0.05 determined by Student's t test.

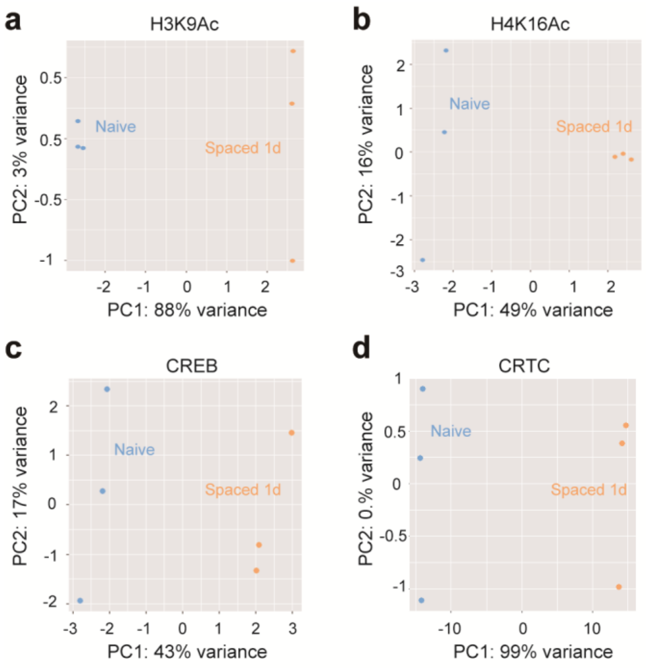

Supplementary Figure 5. Hirano et al.

Supplementary Figure 5 Principal component analysis of the ChIP-seq samples.

The biological replicates in the ChIP-seq data for H3K9Ac **(a)**, H4K16Ac **(b)**, CREB **(c)** and CRTC **(d)** are clustered but well segregated from any samples in different conditions.

The distributions of the sequencing reads were analyzed by a principal component analysis (PCA) in DESeq2. In each cases, the samples of the spaced trained flies (orange dots) were clustered separately from those of the naïve control flies (blue dots).

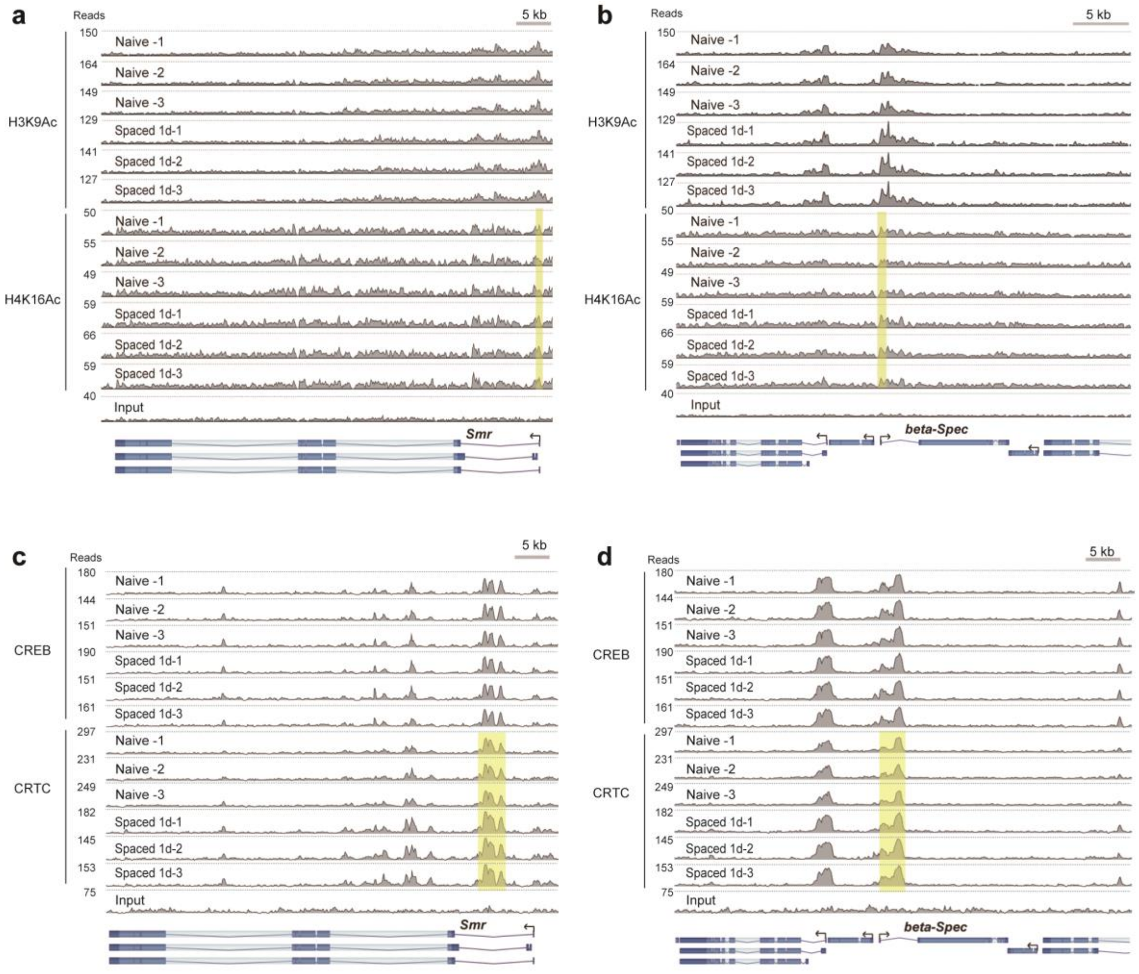

Supplementary Figure 6. Hirano et al.

Supplementary Figure 6 The representative ChIP-seq signals at the regions with increase in CREB/CRTC and histone acetylation.

The chromosome views of the same ChIP-seq data as in Fig. 3e and j were shown. The increase in histone acetylation and CRTC binding identified in this study were highlighted by light-yellow vertical bars. The y axes show the number of the mapped reads, and the upper limits were adjusted to the number of the total reads in each samples. The base line indicates zero read.

(a, c) The region near *Smr*.

(b d) The region near *beta-Spec*.

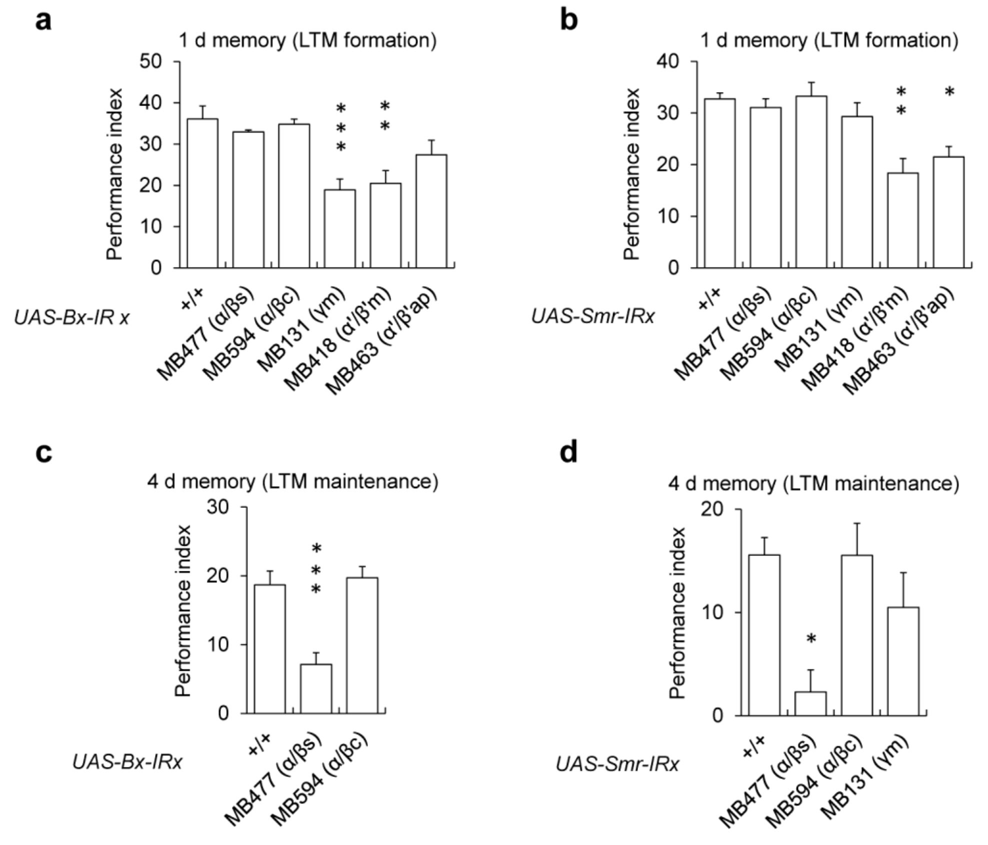

Supplementary Figure 7 Hirano et al.

Supplementary Figure 7 *Bx* and *Smr* are required in the α/βs neurons for 4-day LTM maintenance.

(a, b) Knockdown of *Bx* in the α/βs, α/βc and α'/β'ap neurons doesn't affect LTM formation, neither does knockdown of *Smr* in the α/βs, α/βc and γm neurons (**a**, from left to right, n = 8, 8, 8, 8, 10 and 8; **b**, n = 8, 12, 12, 8, 8 and 8).

(c, d) Knockdown of *Bx* and *Smr* in the α/βs neurons impairs 4-day LTM maintenance (**c**, from left to right, n = 8, 12 and 11; **d**, n = 8, 8, 12 and 12).

Wild-type flies (+/+) or the transgenic flies carrying each *UAS-IRs*.were crossed with the split-GAL4 driver lines, named as MB series indicated at the bottom. The neuropils labeled by each split-GAL4 drivers were indicated in parentheses. Their progenies were subjected to

spaced training and 1 day memory (LTM formation) or 4 day memory (4-day memory maintenance) were tested. Data was analyzed by One-way ANOVA followed by Bonferroni testing. *, $p < 0.05$, **, $p < 0.01$, ***, $p < 0.001$.

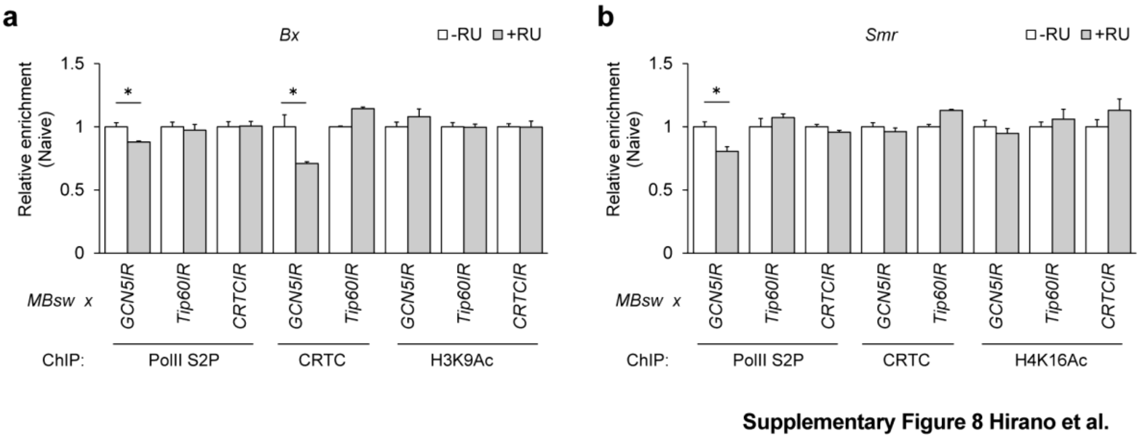

Figure 8 GCN5 regulates expression of *Bx* and *Smr* in naïve flies.

(a, b) PolII S2P bindings at *Bx* and *Smr* are reduced by knockdown of *GCN5,* but are not affected by knockdown of *Tip60* or *CRTC*.

The transgenic UAS-RNAi lines targeting the indicated genes were crossed with MBsw expressing FLAG-KASH. The naïve flies were reared on food with or without RU for 3 days to induce RNAi. The MB nuclei were prepared, and subjected to ChIP assay for PolII S2P, CRTC and H3K9Ac (a) or H4K16Ac (b). (n=3 for all data.). p, < 0.05 determined by Student's t test.

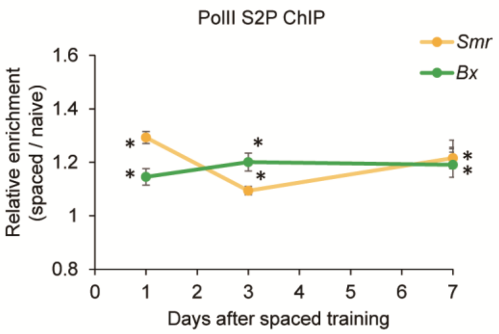

Supplementary Figure 9. Hirano et al.

Supplementary Figure 9 PolII S2P binding to the LTM maintenance genes sustains after spaced training.

The purified MB nuclei expressing FLAG-KASH prepared from naïve control flies or flies at the indicated times after spaced training were subjected to ChIP analysis using anti-PolII S2P antibodies. The ChIP DNA was analyzed at the indicated gene loci. At each time point, the naïve control flies were similarly treated without spaced training, sampled at the same age after eclosion, and used to calculate relative enrichment (n=3 for all data.). At each time point, data of spaced trained flies and naïve flies were compared by Student's t test. *, $p < 0.05$.

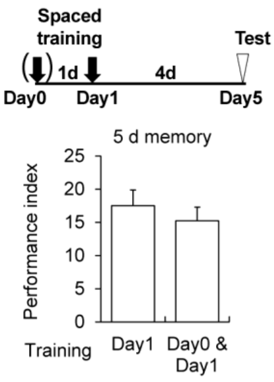

Supplementary Figure 10. Hirano et al.

Supplementary Figure 10 The second round of spaced training does not enhance LTM.

Wild-type flies were subjected to spaced training at Day 0 and Day 1, and tested at Day 5. 4-day memory was analyzed to test LTM, since another long-lasting memory, ARM persists until 4 days after spaced training (n = 8 for both data). Student's t test indicated no significant difference.

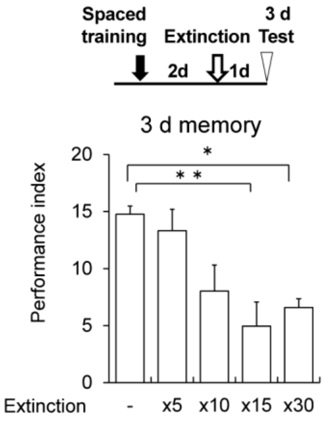

Supplementary Figure 11. Hirano et al.

Supplementary Figure 11 Repeated exposure to the learned odor results in suppression of LTM.

The spaced trained flies were exposed to the learned odor 5, 10, 15 or 30 times (the extinction paradigm) at 2 days after spaced training, resulting in suppression of LTM tested 3 days after spaced training (from left to right, n = 14, 12, 12, 12 and 8; One-way ANOVA; p = 0.0003). *, p < 0.05; **, p < 0.01 by Bonferroni testing.

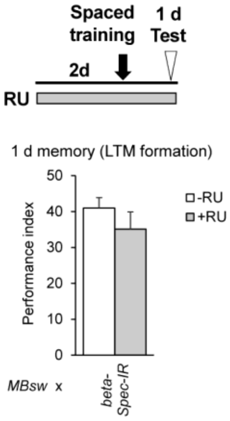

Supplementary Figure 12. Hirano et al.

Supplementary Figure 12 *beta-Spec* is not required for LTM formation.

UAS-beta-Spec-IR flies were crossed with MBsw, and 1-day memory was analyzed as shown (n = 8 for both data). Student's t test indicated no significant difference.
