# Supplementary material for: Shifting transcriptional machinery is required for long-term memory maintenance and modification in Drosophila mushroom bodies
Source: Nat Commun. 2016 Nov 14;7:13471. doi: 10.1038/ncomms13471 (PMC5114576; doi:10.1038/ncomms13471)
Supplement: Peer Review File [file ncomms13471-s13.pdf]

Reviewers' comments:

Reviewer #1 (Remarks to the Author):

This interesting manuscript from Hirano et al investigates the ongoing role of gene transcription for the formation and maintenance of 4-7 day long-term memory. The authors induce the expression of RNAi directed against CREB co-activators and histone acetyl transferases (HATs) in Mushroom Body (MB) intrinsic cells before learning, 1-4 days after learning, or 4-7 days after learning. Their results suggest that the co-activator CRTC and the HATs GCN5 and Tip60 are involved at different times after learning but not during memory formation. Using CHIP-seq they identify learning related target genes of CREB/CRTC and histone acetylation and verify three of them to be functionally involved in memory relevant processes. Interestingly the data suggests that distinct transcription systems are required during the experimentally assessed periods: an "early" phase (1-4d) and a "late" phase (4-7d) to ensure learned behaviour. Furthermore, the authors functionally linked the differences in transcriptional machinery to behaviour by looking at the susceptibility to memory extinction.

The data are certainly interesting and are of sufficient impact to appeal to the readership of Nature Communications. Most of the experiments follow a clear logic and are generally well designed. However, there are some issues with data, the interpretation of the results and the conclusions drawn, which I suggest the authors address before I can recommend publication.

Results:

1) For almost all the experiments the authors use a Gal4 or LexA driver which is known to label functionally heterogeneous sub-populations of Kenyon cells (KCs). Based on their findings the authors propose a shift in transcriptional machinery throughout different phases of memory maintenance. Since, it is well established that discrete KC subpopulations are involved in memory storage/retrieval at different points in time, it might be that there is a shift in memory relevant KC sub-populations over time with memory traces in each one depending on different transcription machinery. This would by no means alter the importance of the findings but it would somewhat alter the conclusions and would mean that the process was not simply shifting between different transcription processes in the same population of MB neurons. The authors should address this point, and either add new data or substantially adjust the manuscript.

2) The authors propose that they are investigating memory maintenance by looking at manipulations applied at different times after learning. However, from the data presented it is not clear how they are able to discriminate between memory maintenance and specific effects on retrieval. This certainly deserves comment since the authors exclusively claim to investigate memory maintenance, rather than memory retrieval. I think this is particularly important to address/comment on because the authors present some very interesting data near the end on the interplay of transcription systems and retrieval induced extinction. One might argue that these data indeed hint more towards a retrieval phenomenon. So again,

the authors should address this issue experimentally or substantially rewrite the manuscript. I am aware that it might be rather difficult to separate these two things experimentally, but that at least justifies an attempt and discussion of the issue.

3) In the section "Epigenetically modified target genes are required for early LTM maintenance" the authors make statements about effects that are not backed up by the statistics. e.g. in Fig 4 and Fig S5 "...we found significant increases in H3K9Ac (Supplementary Fig. 5a), H4K16Ac (Supplementary Fig. 5b), and CRTC binding (Supplementary Fig. 5c) 1 day after spaced, but not massed training." and "...To determine whether increased transcription of LTM maintenance genes requires histone acetylation and CRTC binding, we knocked down GCN5, Tip60 or CRTC, and found that these interventions suppress increased PolII S2P binding 1 day after spaced training (Fig. 4c)." In both cases the statistics presented do not support these claims. For example, in figure 4c-f the authors compare the RU- vs RU+ groups which do not allow one to conclude that the training increases the binding of different proteins (PollS2P, CRTC, H3K9Ac and H4k16Ac). The authors do the same thing in several figures and should either perform the correct statistics for the claim or rewrite the text and adapt the conclusions.

4) The authors should specifically define and name the different phases early in the manuscript and subsequently use the terms throughout. e.g. early LTM maintenance (4d) late LTM maintenance (7d) and memory formation (1d). I would suggest using the day notation rather than the fairly arbitrary 'early/late'.

5) The authors should explain in the Material and Method section why they use two different approaches to normalize the data, "...The counted read numbers in individual regions were normalized by elav for H3K9Ac ChIP-seq, and gapdh2 for H4K16Ac ChIP."

6) The authors should rearrange the figures so that the reader does not have to jump back and forth between the figures as they appear to be cited wthroughout the text.

7) The authors claim to see nuclear translocation of an HA-tagged CRTC in Supplemental 1A. This is a very nice part of the study but I am currently unable to decide whether the data presented really justify the conclusions. The authors should show images with the comparable magnification and resolution of Fig 3b where nuclei can be clearly seen. At present it is not possible to distinguish cell labelling from nuclear. A double stain with a nuclear marker would also help. I also wonder whether then number of nuclei labelled is equivalent to the number estimated to be activated by an odor? If so, this would be consistent with an odor-specific memory-relevant event.

8) the authors in fact use a nuclear stain in Fig 3B and it would be nice if they showed the entire image as only that channel in addition to the merge. The authors also do not say what the magenta channel actually represents/stains.

9) I may be mistaken but I could not see data for CREB and CRTC ChIP-Seq in naïve flies.

10) Page 10. I think some of the validation of targets should be in the main figures rather than all in Supplemental Fig 5.

11) 'our data suggest the possibility that expression of maintenance genes ,ay shift from being CREB-dependent to Bx-dependent'. This would be very interesting. Do the authors know it's in the same cells though? This should be discussed as mentioned above.

Reviewer #2 (Remarks to the Author):

The article by Hirano et al. explores the transcriptional and epigenetic changes that underlie long-term memory maintenance in Drosophila mushroom bodies. More precisely, it presents a number of knockdown experiments in Drosophila using RNAi (or a repressor isoform in the case of CREB activity) followed by behavioral experiments investigating long-term memory (LTM) formation and maintenance. In addition, they performed epigenomic experiments to characterize transcriptional and epigenetic changes occurring during LTM storage in the mushroom bodies (MBs). Based on indirect evidence, they authors concluded that the transcriptional requirements during LTM formation and maintenance differ and pinpoint a number of transcription factors, transcriptional co-activators and target genes that could play important roles in this transition. Although the scope of the study is quite ambitious, important controls and gene expression data are missing, which greatly reduces the significance of the study. I will only comment below the most important caveats.

1. The whole manuscript is based on knock-down experiments for a large number of genes. However, the efficiency and specificity of these manipulations is not proven.

2. RNA levels quantification experiments are absolutely essential for interpreting the results. The authors are supposedly investigating a transcriptional shift without directly assaying transcription even once. The closer they got to investigate transcription are the experiments in which they examined the binding of RNPII-S2P to a few promoters. However, the presence of this phosphorylation does not represent a direct and accurate read out of transcription. Drosophila provides a number of genetic tools that would enable transcriptomics analyses in the same cells used in ChIP-seq experiments. Alternatively, they could profile nuclear RNAs.

3. Although the authors refer to increases in CREB/CRTC binding associated with memory (e.g., page 9), it seems that they only produced profiles 1 day after spaced training (or at least the only presented those profiles in Fig. 3f and Sup. Figures). In the absence of the profiles in naïve animals, the authors cannot make any statement regarding increases or decreases.
Furthermore, ChIP-seq experiments for histone marks were conducted in triplicate, but it seems that in the case of CREB and CRTC profiles they only examined one sample (without duplicates). Is this the case?
Also, the coverage of some ChIP-seq experiments (<8 mo, an in some intances <3 mo) is extremely low.

4. Counted reads in H3K9Ac ChIP-seq were normalized with elav and in H3K9Ac ChIP-seq with gapdh2. It seems that the authors obtained whole-genome information to finally normalize using the values for a single gene. This normalization approach is very unusual and probably a bad idea because it may greatly distorts the results. There are different viable approaches to normalize ChIP-seq experiments, some based on internal parameters, others relying in the inclusion of external DNA (spike-in). The method used by the authors does not seem valid. Why did they use a different "normalizer" for each histone modification? Does the normalizer alter the result? The bias introduced by this approach could easily explain some trends presented in the figures.

5. In page 8 it is indicated that "... we found significant increases in H3K9Ac 1 day after spaced training, ... we found significant increases in H4K16Ac 1 day after spaced training", and it is indicated that in both cases a majority of these regions mapped near TSSs. Based on the scatter plots in Fig. 3 it is obvious that the statements "... we found significant decreases in H3K9Ac and H4K16Ac 1 day after spaced training" would be also true. The decision to focus in regions presenting a 20% increase in acetylation seems arbitrary and directs the conclusions. The authors should perform parallel analyses with those genomic regions presenting a 20% decrease. It is likely that they will also find an enrichment for neuronal genes (since they are dealing with neuronal tissue) and enrichment in the proximity of TSSs (since this is a strong feature of the two marks under analysis).

Points 3, 4 and 5 greatly reduce the confidence on the genomic analyses.

Other problems
6. Fig. 4c-f indicate that interfering with CRTC during memory acquisition prevents the increase in polII-S2P and histone acetylation that the authors propose to be important for memory expression (n fact, they observed a reduction of binding compared to controls). However, Fig 1b earlier showed that interfering with CRTC has no effect in memory formation. How do the authors explain these results?

7. The use of references is not always appropriate. They often refer to articles that did not directly investigate the accompanying statement and completely ignore important controversies regarding the role of CREB or CBP in memory formation.

8. Figures and legends should improve. The brief figure legends make the interpretation of figures very difficult. For example, what is presented in Fig. 3c or 3d? In Fig 3c are the 2 images in the same scale? What is the discontinuous line in the graphs in Fig. 4? Resolution of IHC images in Figures 2a-c is too low.

Reviewer #3 (Remarks to the Author):

The manuscript entitled 'Shifting transcriptional machinery is required for long-term memory maintenance and modification in Drosophila mushroom bodies' analyses

transcriptional and histone acetylation changes during the formation and maintenance of long-term memory (LTM) in Drosophila melanogaster mushroom bodies. The authors find that LTM formation is CREB and CBP dependent, whereas early LTM maintenance requires CREB, CRTC, Tip60, and GCN5. Late maintenance is CREB independent and requires Tip60 and the transcription factor Beadex (Bx). The authors then identify CREB/CRTC target genes that are required for maintenance and extinction and link extinction to the CREB/CRTC-dependent memory phase.
Overall this study is of high interest to the community and I personally like it a lot. Unfortunately it also suffers from several shortcomings, some major and some minor, which in my view should be addressed prior to publication.

Major concerns:
1. Unfortunately I have to start with a purely technical matter of high relevance for the data interpretation. The authors perform ChIP-seq experiments for H3K9ac and H4K16ac and compare naïve to spaced training-exposed animals and base a large part of their subsequent findings on the analysis of this data. This analysis is in many parts not of high quality, in some cases also flawed. I'll enumerate the most prominent issues. First of all, I would expect a proper quality control of ChIP-seq data, something that is not supplied by the authors. I will not go into the details of the QC and rather recommend reading a recent publication in the field of learning and memory (PMID: 26656643). I am not too happy with the peak calling and the subsequent differential analysis performed, especially the windowing to 400 bases that is not followed by a proper merging of contiguous enriched regions. Although I consider the above-mentioned problems of importance, these are all minor issues when compared to the lack of multiple testing correction for the significance calculations of differential 'peaks' (naïve vs. trained). Why don't the authors use statistics based on a negative binomial distribution (reads are not normally distributed) and a proper FDR estimation as for example implemented in the DESeq2 package? In the end, I fear that a proper analysis of the ChIP-seq data might change large parts of the manuscript, although I hope that the main findings persist. Along the lines, I would like to point out that the visualization of the ChIP-seq data is sub par, i.e. missing axes and scales in the browser pictures.
2. I'll have to start with another 'unfortunately', as parts of the novelty of the manuscript has been addressed in a recent study (PMID: 26656643). Especially the part of changes in regulatory networks between formation and LTM is described and should be adequately cited. In consequence, many parts of the manuscript need to be reformulated, from the summary to the discussion. Again, this is not the authors fault but still needs to be addressed appropriately.
3. Large parts of the manuscript are hinged on CRTC's central regulatory function in memory maintenance (CREB/CRTC in maintenance and extinction). Why then did the authors do not perform proper CRTC ChIP-seq experiments with at least two replicates, before and after learning (currently n=1 and no comparison to naïve state). Changes in acetylation are usually small and relatively hard to detect whereas TF or co-regulator changes are much stronger (e.g. PMID: 20393465 and the follow up manuscript). Given that the authors have CREB-IR ChIP-qPCR data it would be great if they could supply sequencing results.

Minor concerns:
1. The method for cell type-specific extraction of nuclei is not novel, it is just used in D.m. mushroom bodies for the first time. This hardly qualifies as a novel method and several sentences in the manuscript should be changed, in consequence.
2. I am a little worried with the amount of performance index variation in the -RU cases. In many cases the -RU performance in - (wt? Animals) is lower than for the transgenes, sometimes up to several PI points (e.g. Fig. 1A, CRTC-IR -RU vs. - -RU). Actually, many of the significant changes are in part due to elevated baseline levels of memory performance (e.g. Fig1B, CREB2b -RU vs. - -RU). Are these fluctuations normal or is it due to some special treatment? (see also Fig. 4a...)
3. Since the authors analyze histone acetylations in the context of LTM formation it would have been nice to reflect their findings in light of a recent very nice review of the Barco laboratory (PMID: 25269450).
4. Why is the purity of the IP-ed nuclear fractions only at 90%? Given previous publications I would have expected at least 95%.
5. It would be great to have some 'enrichment' assessment for the overlap of CRTC-H3K9ac-H4K16ac (using FDRs as described above). The authors can use a permutation-based approach or hypergeometric test to estimate if this overlap is non-random.
6. I am really not sold on the GO analysis in Fig.3i. Showing three terms, two of which have not much to do with learning processes is not very convincing. The figure is also not very appealing but this is a negligible problem.
7. What is the rationale for only looking at genes with increased acetylation upon learning. Wouldn't the authors miss out on all negative regulators, including e.g. repressors and miRNAs? Why disregard this information?
8. Would be great if the authors could recheck their numbers. I just quickly checked the number of CREB/CRTC genes (1396 instead of 1394) and the ones with increasing H3K9ac (348 entries instead of 346). Seems like a systematic error... (cat <input_file> | cut -f4 | sort -u | wc -l)
9. In the section 'Epigenetically modified target genes are required for early LTM maintenance' the authors short-list 20 genes, all epigenetic regulators and other 'representative genes'. Could you please explain how you got to these 'representative genes', as I couldn't find an explanation in the material and methods section. Also, isn't it interesting that none of the selected chromatin modifiers showed any impact on LTM maintenance?
10. I am more than a little intrigued by the finding that CREB/CRTC target genes show acetylation changes in a memory formation paradigm (Fig. 4b and c). CRTC, Tip60, and Gcn5 have no effect on memory formation (Fig. 1) yet they change the acetylation and polII occupancy of memory maintenance target genes in a memory formation paradigm. Could you please also compare the PolII, CRTC, and acetylation levels to the naïve state? Along the lines, why does Fig. 4F only contain Smr levels?
11. It would be great if the authors re-word some of their statements. For one, the authors use the word 'epigenetic regulators' to specify a set of 'chromatin modifiers'. Epigenetic regulation far exceeds chromatin modifications (e.g. miRNAs...). I would also like to stress the point that HATs do have other targets than histones, among them many transcription factors (e.g. Gcn5). Attributing LTM performance changes purely to possible histone PTM

changes is a very simplistic view. A nice treatment of this fact can be found in the above-mentioned review by Barco (PMID: 25269450).

Responses to Reviewer #1:

We appreciate the valuable comments from Reviewer #1. In our revised manuscript, we fully addressed all the issues raised by Reviewer #1. All of these helped substantially strengthen our revised manuscript.

Our point to point responses to the comments of Reviewer #1 are as follows.

1) For almost all the experiments the authors use a Gal4 or LexA driver which is known to label functionally heterogeneous sub-populations of Kenyon cells (KCs). Based on their findings the authors propose a shift in transcriptional machinery throughout different phases of memory maintenance. Since, it is well established that discrete KC subpopulations are involved in memory storage/retrieval at different points in time, it might be that there is a shift in memory relevant KC sub-populations over time with memory traces in each one depending on different transcription machinery. This would by no means alter the importance of the findings but it would somewhat alter the conclusions and would mean that the process was not simply shifting between different transcription processes in the same population of MB neurons. The authors should address this point, and either add new data or substantially adjust the manuscript.

To address this comment, we used split-GAL4 drivers, and found that *CRTC* (Sup. Fig. 3b, d), *Bx* (Sup. Fig. 9a, c) and *Smr* (Sup. Fig. 9b, d) were required in one particular subpopulation of KC, called the α/β surface (α/βs) KCs for 4-day LTM, indicating that the transcription shift for LTM maintenance likely occurs within the α/βs KCs. Unfortunately, the split-GAL4 driver is not designed to acutely induce RNAi, and thus could not exclude developmental defects (Sup. Fig. 3f, g; l.147-151) and the possibility that other subpopulations of KCs may contribute to transcriptional shifts during 4-7 day LTM maintenance. We mentioned this in Discussion (l.343-353).

2) The authors propose that they are investigating memory maintenance by looking at manipulations applied at different times after learning. However, from the data presented it is not clear how they are able to discriminate between memory maintenance and specific effects on retrieval. This certainly deserves comment since the authors exclusively claim to investigate memory maintenance, rather than memory retrieval. I think this is particularly important to address/comment on because the authors present some very interesting data near the end on the interplay of transcription systems and retrieval induced extinction. One might argue that these data indeed hint more towards a retrieval phenomenon. So again, the authors should address this issue experimentally or substantially rewrite the manuscript. I am aware that it might be rather difficult to separate these two things experimentally, but that at least justifies an attempt and discussion of the issue.

The reviewer pointed out that identified transcriptional machineries could be required for memory retrieval, rather than memory maintenance. Unfortunately, we could not separate these two experimentally at the moment. Therefore, while we propose model that our identified transcriptional machineries are required for LTM maintenance, we mentioned the possibility in the discussion as state "Currently, we propose that the alterations in histone acetylation and transcription we uncovered are required for memory maintenance. However, we note that decreases in memory after formation could be caused by defects in retrieval as well as maintenance. Thus, if recall of LTM requires changes in transcription, and if recall at different times after training require different transcriptional changes, it remains possible that the epigenetic and transcriptional changes we found are required for recall and not maintenance." (l.354-360)

The reviewer raised an additional interesting view regarding Fig. 7d, in which activation of CRTC (CRTC-SA expression) allowed LTM extinction when LTM is not normally extinguishable. As the reviewer concerned, CRTC-SA may simply enhance LTM retrieval during the extinction protocol, which could reasonably induces LTM extinction. If this is the case, *beta-Spec*, the CRTC-downstream gene important for LTM extinction should be required for LTM retrieval. However, *beta-Spec* was not involved in LTM retrieval at any time point we examined (Fig. 4d, Sup. Fig. 14). Thus, the CRTC- beta-Spec pathway in LTM extinction would not be related to LTM retrieval.

3) In the section "Epigenetically modified target genes are required for early LTM maintenance" the authors make statements about effects that are not backed up by the statistics. e.g. in Fig 4 and Fig S5 "...we found significant increases in H3K9Ac (Supplementary Fig. 5a), H4K16Ac (Supplementary Fig. 5b), and CRTC binding (Supplementary Fig. 5c) 1 day after spaced, but not massed training." and "...To determine whether increased transcription of LTM maintenance genes requires histone acetylation and CRTC binding, we knocked down GCN5, Tip60 or CRTC, and found that these interventions suppress increased PolII S2P binding 1 day after spaced training (Fig. 4c)." In both cases the statistics presented do not support these claims. For example, in figure 4c-f the authors compare the RU- vs RU+ groups which do not allow one to conclude that the training increases the binding of different proteins (PollS2P, CRTC, H3K9Ac and H4k16Ac). The authors do the same thing in several figures and should either perform the correct statistics for the claim or rewrite the text and adapt the conclusions.

In response to reviewer's criticism, we carefully checked and amended the text to be supported by statistic analyses.

4) The authors should specifically define and name the different phases early in the manuscript and subsequently use the terms throughout. e.g. early LTM maintenance (4d) late LTM maintenance (7d) and

memory formation (1d). I would suggest using the day notation rather than the fairly arbitrary 'early/late'.

As the reviewer suggested, we changed ‘early LTM maintenance’ to ‘4-day LTM maintenance’, and likewise ‘late LTM maintenance’ to ‘7-day LTM maintenance’.

5) The authors should explain in the Material and Method section why they use two different approaches to normalize the data, "...The counted read numbers in individual regions were normalized by elav for H3K9Ac ChIP-seq, and gapdh2 for H4K16Ac ChIP."

The reviewer 2 and 3 also raised the same issue. To address this issue, we reanalyzed the ChIP-seq data using the Bioconductor package DESeq2 which was recommended by the reviewer 3 (Methods, l.621-635). Instead of using a particular gene as an internal control, DESeq2 provides fold change and adjusted p value (FDR) between conditions, based on negative binominal generalized linear models (Love et al., Genome Biol, 2014). Thus all the results in the revised manuscript should be reliable based on the proper data normalization and statistics.

6) The authors should rearrange the figures so that the reader does not have to jump back and forth between the figures as they appear to be cited throughout the text.

As suggested by the reviewer, we have rearranged panels and contents especially in Figs 1 and 2 to avoid irregular citations.

7) The authors claim to see nuclear translocation of an HA-tagged CRTC in Supplemental 1A. This is a very nice part of the study but I am currently unable to decide whether the data presented really justify the conclusions. The authors should show images with the comparable magnification and resolution of Fig 3b where nuclei can be clearly seen. At present it is not possible to distinguish cell labelling from nuclear. A double stain with a nuclear marker would also help. I also wonder whether then number of nuclei labelled is equivalent to the number estimated to be activated by an odor? If so, this would be consistent with an odor-specific memory-relevant event.

We have added the high magnification images showing CRTC nuclear translocation in Fig. 1 and Sup. Fig. 1, in accordance with the reviewer’s suggestion. We also counted the nuclei with strong CRTC signals relative to CRTC signals in the Calyx (the dendritic region), and found that approximately 4% of KCs showed nuclear CRTC (Fig. 1m, Sup. Fig. 1d). As the reviewer expected, this number is consistent with previously

reported populations of neurons that exhibited electrophysiological responses to an odor (6 ± 5% in Turner et al., J Neurophysiol. 2008, 99:734-46). We have mentioned these in Results (l.119-122).

8) The authors in fact use a nuclear stain in Fig 3B and it would be nice if they showed the entire image as only that channel in addition to the merge. The authors also do not say what the magenta channel actually represents/stains.

As the reviewer suggested, we added nuclear staining images for all relevant panels in the figures (Fig 1d, e, g, h, j, k; Fig 3b; Sup Fig. 1a, b). We also explicitly indicate that magenta channel represents fluorescent signals of TO-PRO-3 iodide or propidium iodide, which specifically stains the nucleus, in the figure legends (Fig. 1, 3, Sup. Fig. 1).

9) I may be mistaken but I could not see data for CREB and CRTC ChIP-Seq in naïve flies.

We added the ChIP-seq data comparing naïve and trained flies with three biological replicates (Fig. 3j). This analysis revealed that CREB binding is unchanged in naïve and trained flies, but CRTC binding is elevated in the trained flies (Sup. Table 7). We have revised the text to include these results (l.188-195).

10) Page 10. I think some of the validation of targets should be in the main figures rather than all in Supplemental Fig 5.

As the reviewer suggested, we have moved data in the previous Supplemental Fig. 5 to new main figures (Fig. 4a, c, 5a, b).

11) 'our data suggest the possibility that expression of maintenance genes may shift from being CREB-dependent to Bx-dependent'. This would be very interesting. Do the authors know it's in the same cells though? This should be discussed as mentioned above.

As the reviewer recommended, we discussed whether the transcriptional shift from CREB/CRTC to Bx occurs in the same neurons (l.343-353). Although we could not fully exclude the possibility that CREB/CRTC- and Bx-dependent transcription may occur in different sets of KC neurons, the shift in transcription likely occur in the same neurons based on our observations that 1) our new data using split-GAL4 drivers indicate that CRTC and Bx are required in the α/βs KCs (Sup. Fig. 3d, 9c), 2) CREB/CRTC binds to the *Bx* gene locus (Fig 3j), and 3) *Bx* expression initially requires CRTC (Fig. 5c).

Responses to Reviewer #2:

We thank Reviewer #2 for the full assessment of our work, for pointing out the major weaknesses in our study, and also for the suggestion of critical experiments and analysis that have helped to significantly improve the validity and the strength of our findings. In the revised manuscript, we have included a number of control experiments including validation of gene knockdown and of ChIP-Seq data. We believe that our data have substantially improved and have become much more convincing.

Followings are our point to point responses to the comments of Reviewer #2.

1. The whole manuscript is based on knock-down experiments for a large number of genes. However, the efficiency and specificity of these manipulations is not proven.

To address the efficiency, using the heat-shock GAL4 driver, we showed that each RNAi construct significantly reduced expression of target genes (Suppl. Fig. 2). To address the specificity, we examined all possible off-target genes. The off-target effects could appear if any of 19-mer or more in the RNAi sequence shows a perfect match to other genes (Kulkarni M., et al, 2006, Nature Method, vol3, p833-838). Based on this criterion, we found that expression of those possible off-target genes were not affected (Sup. Fig. 2), suggesting that the RNAi lines we used are highly specific.

2. RNA levels quantification experiments are absolutely essential for interpreting the results. The authors are supposedly investigating a transcriptional shift without directly assaying transcription even once. The closer they got to investigate transcription are the experiments in which they examined the binding of RNPII-S2P to a few promoters. However, the presence of this phosphorylation does not represent a direct and accurate read out of transcription. Drosophila provides a number of genetic tools that would enable transcriptomics analyses in the same cells used in ChIP-seq experiments. Alternatively, they could profile nuclear RNAs.

As pointed out by the reviewer, we quantified RNA, in addition to binding of RNA polymerase II-S2P (RNPII-S2P). Nuclear RNA were prepared from isolated MB nuclei (Fig. 4b). Our analysis revealed that, consistent to the RNPII-S2P data (Fig. 4a), nuclear RNA transcripts of the selected candidate genes were mostly increased at 1d after spaced training, except *trx*. These results strongly support the validity of the RNPII-S2P ChIP assay to monitor the transcriptional activities. We speculate reasons for the failure to detect increases in nuclear RNA of *trx* either because *trx* expression may be more transiently increased in a specific time window, or decay of *trx* nuclear RNA may be more rapid than other nuclear RNAs. These interpretations were mentioned in the Result (l.226-227).

As the reviewer suggested, we sought to profile nuclear RNAs using the RNA-seq method. However, we faced the technical problem using FLAG-KASH tag, in which we could isolate the fixed nuclei, but not unfixed nuclei, although the similar method reported in mice using SUN1-sfGFP-Myc (Mo, A., et al. Neuron 2015) allowed isolation of the unfixed nuclei. This is probably due to the difference in nuclear-tagging. The RNA preparation from the fixed nuclei required recovery of crosslinking by incubation at 65℃ for 6 h, which could affect the RNA profiles. Nonetheless, in the future study, we are eager to try nuclear RNA profiling and also try the genetic methods, particularly one of the most applied methods, using GFP-tagged ribosomal protein (UAS-GFP::RpL10A) (Thomas, A. et al, PLOS ONE, 2012), as suggested by the reviewer.

3. Although the authors refer to increases in CREB/CRTC binding associated with memory (e.g., page 9), it seems that they only produced profiles 1 day after spaced training (or at least the only presented those profiles in Fig. 3f and Sup. Figures). In the absence of the profiles in naïve animals, the authors cannot make any statement regarding increases or decreases.
Furthermore, ChIP-seq experiments for histone marks were conducted in triplicate, but it seems that in the case of CREB and CRTC profiles they only examined one sample (without duplicates). Is this the case?
Also, the coverage of some ChIP-seq experiments (<8 mo, an in some intances <3 mo) is extremely low.

As the reviewer concerned, we have previously showed CREB and CRTC binding profile without duplicates. We added new analysis with biological three replicates comparing naïve flies and spaced trained flies revealed that, although CREB binding is unchanged, CRTC binding to all CREB/CRTC binding sites are increased after spaced training as compared to the naïve controls (Fig. 3j, Sup. Table 7).

The reviewer also concerned about the low coverage of our ChIP-seq data. We therefore performed the saturation correlation analysis using the MEDIPS R package, asking whether or not the sequencing was deep enough to reproduce the data. While we admit that the coverage rates of our ChIP data are rather low, the results of MEDIPS revealed high correlation in all samples (more than 0.85), except input samples (0.64 and 0.76), suggesting that our ChIP-seq data is deep enough to get reproducible results. We included these validations in the Method section in the revised manuscript (l.590-595).

4. Counted reads in H3K9Ac ChIP-seq were normalized with elav and in H3K9Ac ChIP-seq with gapdh2. It seems that the authors obtained whole-genome information to finally normalize using the values for a single gene. This normalization approach is very unusual and probably a bad idea because it may greatly distorts the results. There are different viable approaches to normalize ChIP-seq experiments, some based on internal parameters, others relying in the inclusion of external DNA (spike-in). The method used by the authors does not seem valid. Why did they use a different "normalizer" for each histone modification? Does the

normalizer alter the result? The bias introduced by this approach could easily explain some trends presented in the figures.

To address this concern, we newly introduced the DESeq2 package, as suggested by the reviewer #3, to analyze the ChIP-seq raw data, in which negative binominal generalized linear models are applied without assuming specific genes as internal control. The proper statistics in DESeq2 greatly reduced the number of the candidate genes (Fig. 3k). Half of the behaviorally tested 20 genes were retained, and thus we analyzed only these 10 genes further. We believe that the result presented in the revised manuscript now become much more trustable and reliable based on the proper normalization and statistics.

5. In page 8 it is indicated that "... we found significant increases in H3K9Ac 1 day after spaced training, .. we found significant increases in H4K16Ac 1 day after spaced training", and it is indicated that in both cases a majority of these regions mapped near TSSs. Based on the scatter plots in Fig. 3 it is obvious that the statements "... we found significant decreases in H3K9Ac and H4K16Ac 1 day after spaced training" would be also true. The decision to focus in regions presenting a 20% increase in acetylation seems arbitrary and directs the conclusions. The authors should perform parallel analyses with those genomic regions presenting a 20% decrease. It is likely that they will also find an enrichment for neuronal genes (since they are dealing with neuronal tissue) and enrichment in the proximity of TSSs (since this is a strong feature of the two marks under analysis).

The reviewer criticized that we should analyze the decreased regions in H3K9Ac and H4K16Ac. We thus determined decrease in histone acetylation, and as the reviewer expected, decrease in H3K9Ac and H4K16Ac occurred in the proximity of the TSSs (Fig. 3i). We found that the clustered GO terms obtained from the H3K9Ac- and H4K16Ac-decreased genes were related to the neuronal functions and the energy metabolism, respectively (Sup. Table 8). Energy metabolism would also be related to the neural activities, and therefore the enriched GO terms would support the relevance of our study.

Other problems

6. Fig. 4c-f indicate that interfering with CRTC during memory acquisition prevents the increase in polII-S2P and histone acetylation that the authors propose to be important for memory expression (in fact, they observed a reduction of binding compared to controls). However, Fig 1b earlier showed that interfering with CRTC has no effect in memory formation. How do the authors explain these results?

CRTC is involved in gene expression 1 day after spaced training (Fig. 5c, d), but not 1 d memory performance (Fig. 1b). Importantly, the genes regulated by CRTC are LTM maintenance genes (*Bx* and *Smr*),

which are not required for 1-day memory performance. (Fig. 4e). Therefore, we propose that ongoing transcription 1 day after the training through CREB/CRTC and HATs is essential, not for 1-day memory performance, but for the memory performance at subsequent days (4 days after the training, Fig. 1a, 2a). We included this in Result (l.254-257).

7. The use of references is not always appropriate. They often refer to articles that did not directly investigate the accompanying statement and completely ignore important controversies regarding the role of CREB or CBP in memory formation.

To address this issue, we have checked all the references and citations throughout the text, and corrected citations to appropriately support the content of statements. The reviewer also asked to mention the controversies regarding the role of CREB and CBP in memory formation. In response to this point, we cited two papers from Barco laboratory and from Brindle laboratory, suggesting that the role of the HAT activity of CBP (or even histone acetylation) in memory is not conclusive, but rather its binding activity to various proteins could be essential for LTM formation (l.51; l.384-389).

8. Figures and legends should improve. The brief figure legends make the interpretation of figures very difficult. For example, what is presented in Fig. 3c or 3d? In Fig 3c are the 2 images in the same scale? What is the discontinuous line in the graphs in Fig. 4? Resolution of IHC images in Figures 2a-c is too low.

To address this criticism, we have substantially expanded the figure legends and modified figures. The procedure describing Fig. 3c and the scales in Fig. 3c were added. The discontinuous line in Fig. 4 was for easy comparison of data in the previous manuscript, but it became dispensable now due to reduction in sample sizes, so that we eliminated the line. Also, as the reviewer suggested, we added high resolution images in Fig. 1e, h and k (Fig. 2 in the previous manuscript).

Responses to Reviewer #3:

We are extremely grateful to the reviewer for pointing out many drawbacks including the lack of quality controls and inappropriate procedures in bioinformatics in our previous manuscript. We have followed all the reviewer's recommendation/suggestions, all of which, we believe, significantly increase and strengthen the quality of this work. The point-to-point responses are detailed below.

Major concerns:

1. Unfortunately I have to start with a purely technical matter of high relevance for the data interpretation. The authors perform ChIP-seq experiments for H3K9ac and H4K16ac and compare naïve to spaced training-exposed animals and base a large part of their subsequent findings on the analysis of this data. This analysis is in many parts not of high quality, in some cases also flawed. I'll enumerate the most prominent issues. First of all, I would expect a proper quality control of ChIP-seq data, something that is not supplied by the authors. I will not go into the details of the QC and rather recommend reading a recent publication in the field of learning and memory (PMID: 26656643). I am not too happy with the peak calling and the subsequent differential analysis performed, especially the windowing to 400 bases that is not followed by a proper merging of contiguous enriched regions. Although I consider the above-mentioned problems of importance, these are all minor issues when

compared to the lack of multiple testing correction for the significance calculations of differential 'peaks' (naïve vs. trained). Why don't the authors use statistics based on a negative binomial distribution (reads are not normally distributed) and a proper FDR estimation as for example implemented in the DESeq2 package? In the end, I fear that a proper analysis of the ChIP-seq data might change large parts of the manuscript, although I hope that the main findings persist. Along the lines, I would like to point out that the visualization of the ChIP-seq data is sub par, i.e. missing axes and scales in the browser pictures.

Based on the reviewer's advice and the recommended paper (Halder et al., Nat Neurosci, 19:102-110, 2016; PMID: 26656643), we reanalyzed our ChIP-Seq data in the whole part of our work.

Below, we describe summaries of our reanalysis to show quality control of our ChIP-Seq data in seven parts, which were described in Methods (l.555-637).

1-1) Base quality data

The base qualities were assessed depending on the sequencing system (see below, 1-1)-A and 1-1)-B). In short, we confirmed that the reads pass the FastQC criteria of "per base sequence quality", "Sequence Duplication Levels", "Overrepresented sequences", and "Adapter Content" (Sup. Table 1). Probably

because the ChIP libraries are inherently biased in their sequence composition, some test elicited the warning or errors. Nonetheless, those didn't affect the mapping efficiency (Sup. Table 2).

1-1)-A: CREB/CRTC ChIP-seq:

The sequencing reads for CREB/CRTC were obtained using Miseq, and analyzed by FastQC (Sup. Fig. 6 and Sup. Table 1). The reads were further filtered by the default parameter of CLC Genomics Workbench (cut-off less than the quality value 14, length cut-off less than 16 bp). Then the reads were aligned on the reference genome, in which the mapping efficiency was 77-87% (Sup. Table 2).

1-1)-B: Histone acetylation ChIP-seq:

The sequencing reads for histone acetylation were obtained by the SOLiD system. Due to the colorspace-coding in the SOLiD system which estimates quality value based on the 'color quality', the original raw reads didn't pass 'per base sequence quality'. After the alignment, the base quality of the mapped reads was recalculated from the quality value by Bioscope, which passed the base quality criteria in FastQC (Sup, Fig. 5 and Sup. Table 1). The mapping efficiency was 73-93% (Sup. Table 2).

1-2) Evaluation of sequencing depth

We performed the saturation correlation analysis using the MEDIPS R package to test whether the mapped reads were deep enough to reproduce the data. The estimated correlation was more than 0.85 in all samples, except input samples (0.64 and 0.76), suggesting that our ChIP-seq data were generally deep enough to obtain reproducible results. We added this information in Methods (l.590-595).

1-3) Correlation among biological replicates

We used the MEDIPS R package for the Pearson correlation to evaluate the biological replicates using the mapped reads. Using our ChIP datasets, the Pearson correlation was calculated to be more than 0.9 in any pairs of biological replicates, indicating fairly good reproducibility among the biological replicates. We have mentioned these procedures in Methods (l.596-598). In addition, we performed the principal component analysis (PCA) on distribution of the read counts using DESeq2 (Sup. Fig. 7). The read counts used in PCA were obtained by the windowing described below (1-5). This analysis reveals that each sample in the same experimental condition is fairly clustered but well segregated from any samples in different conditions. These results confirm the validity of the biological replicates and the reproducibility of the subsequent bioinformatics results.

1-4) Aggregate gene plot of histone acetylation

We have calculated and included aggregate gene plots showing that the reads are mapped nearby the TSSs (Fig. 3f, g), using the ngs.plot software, in accordance with the recommended paper by the reviewer (Halder et al., Nat Neurosci, 19:102-110, 2016). These plots confirmed the relevance of the data.

1-5) The window for the read count comparison

In the analysis of histone acetylation in the previous manuscript, we first pooled all peaks called in three replicates excluding the overlapping peaks, and then created the 400 bp windows centering the called peaks. We now realize that this procedure was inappropriate as pointed out by the reviewer. Therefore, in the revised manuscript, we adopted a proper merging of the contiguous window to avoid duplications in the analysis, according to the reviewer's suggestion. In our reanalysis, we have optimized the window parameters because the intergenic regions are small in *Drosophila* (TSSs of the 5692 genes were located within 1000 bp) and simple merging of the window ends up with analyzing two closely-located TSS in one window, thereby reducing the sensitivity of the analysis. Thus, we first trimmed the overlapping regions in the adjacent windows, and if the trimmed windows were less than 200 bp, the windows were merged. These information was described in Methods (l.621-625). Using this window setting, we counted the mapped reads and analyzed by DESeq2 as follows. In the case of analysis of CREB/CRTC ChIP-seq, we used the 1 kb windows covering the entire genome, without overlaps, and the counted reads in each windows were analyzed by DESeq2 (Method, l.629-635).

1-6) Correction for multiple comparisons

We reanalyzed our data using DESeq2, and provide a proper FDR estimation, indicated as adjusted p value (Sup. Table 3-7). As the reviewer anticipated, this proper statistics with DESeq2 greatly reduced the number of the H3K9Ac target genes from 1766 to 633, and the H4K16Ac target genes from 1320 to 182 (Fig. 3k). Fortunately, we found that half of the behaviorally tested 20 genes in the previous manuscript were preserved, which we present in the revised manuscript. The result obtained in the revised manuscript should therefore become much more reliable based on the proper statistics.

1-7) Scales in the browser pictures

We have corrected the visualization of ChIP-Seq data (Fig. 3e, j and Sup. Fig. 8) by adding the axes and the number indicating the scales in accordance with the reviewer's comments..

2. I'll have to start with another 'unfortunately', as parts of the novelty of the manuscript has been addressed in a recent study (PMID: 26656643). Especially the part of changes in regulatory networks between formation and LTM is described and should be adequately cited. In consequence, many parts of the

manuscript need to be reformulated, from the summary to the discussion. Again, this is not the authors fault but still needs to be addressed appropriately.

As the reviewer suggested, we cite the work conducted by Halder et al (2016) (PMID: 26656643). In particular, Halder et al reported that, in mice, DNA methylation is a prominent indicator of gene expression related to memory, but various histone modifications, including H3K9Ac, are rather decoupled to gene expression. Interestingly, our results suggest that histone acetylation could be coupled to gene expression in flies. We think that such species differences would be reasonably explained by the fact that flies lack DNA methylation mechanisms and therefore histone acetylation in flies could have much more significant roles in gene expression. We have included these discussion in the text (l.376-381).

3. Large parts of the manuscript are hinged on CRTC's central regulatory function in memory maintenance (CREB/CRTC in maintenance and extinction). Why then did the authors do not perform proper CRTC ChIP-seq experiments with at least two replicates, before and after learning (currently n=1 and no comparison to naïve state). Changes in acetylation are usually small and relatively hard to detect whereas TF or co-regulator changes are much stronger (e.g. PMID: 20393465 and the follow up manuscript). Given that the authors have CREB-IR ChIP-qPCR data it would be great if they could supply sequencing results.

As the reviewer suggested, we performed ChIP-seq analysis for CREB and CRTC with 3 biological replicates from naïve and spaced trained flies. This revealed that, although CREB binding was not altered except a few regions, CRTC binding was mostly increased after spaced training (Fig 3j, Sup. Fig. 8, Sup. Table 7). The reviewer also asked to show CRTC ChIP-seq data when CREB is knocked-down (CREB-IR). However, as far as we tested, expression of *CREB-IR* was not effective, so that we couldn't conducted this experiment.

Minor concerns:

1. The method for cell type-specific extraction of nuclei is not novel, it is just used in D.m. mushroom bodies for the first time. This hardly qualifies as a novel method and several sentences in the manuscript should be changed, in consequence.

As the reviewer pointed out, we eliminated the term 'novel' to describe the method, and amended all the statement throughout the manuscript (l.158).

2. I am a little worried with the amount of performance index variation in the -RU cases. In many cases the -RU performance in - (wt? Animals) is lower than for the transgenes, sometimes up to several PI points (e.g.

Fig. 1A, CRTC-IR -RU vs. - -RU). Actually, many of the significant changes are in part due to elevated baseline levels of memory performance (e.g. Fig1B, CREB2b -RU vs. - -RU). Are these fluctuations normal or is it due to some special treatment? (see also Fig. 4a...)

We understand the reviewer's concern about the fluctuation in the behavioral data. While we are confident that the difference in performance index is reliably and reproducibly detectable when two groups are compared simultaneously, absolute performance indices are not always stable even in the same group when data were taken on different days. This is common in the fly memory research field. In fact, the levels of the performance index could be greatly affected by slight changes in the environment, such as temperature and humidity, of the behavioral test apparatus and room. We make great efforts to keep the room temperature constant as well as control the humidity of our equipment within 1% accuracy in the behavioral test-room. However, outside of the room, where we keep flies, the temperature and humidity relatively fluctuates. This is why we always perform a side-by-side test of two groups of the same genotype treated with RU or none at the same time. We briefly mentioned this in the Method section (l.710-712).

3. Since the authors analyze histone acetylations in the context of LTM formation it would have been nice to reflect their findings in light of a recent very nice review of the Barco laboratory (PMID: 25269450).

As the reviewer suggested, we cite the review article written by Lopez-Atalaya and Barco (2014). In Introduction, we briefly mentioned their debate over HAT activity of CBP (l.51), and in Discussion, we added their notion in which histone acetylation may not be a cause of gene expression, but rather it might be a passive mark of gene expression (l.384-387).

4. Why is the purity of the IP-ed nuclear fractions only at 90%? Given previous publications I would have expected at least 95%.

The reviewer questioned about the purity of our MB nuclei, which we estimated about 90 %, although 95% purity was obtained in mice using similar methods (Mo, A. et al. Neuron, 2015, 86, 1369–1384). This is probably due to different methods to estimate the purity. While Mo, A. et al. directly visualized and counted GFP-positive nuclei after nuclear isolation, we quantitated the precipitated DNA using qPCR, from the tagged flies with FLAG-KASH, and from the untagged flies as a negative control. Although this properly quantifies the amount of immunoprecipitated nuclei, we could have underestimated the purity. We have assumed the same amount of the background precipitates in the tagged and untagged samples, however, the FLAG-KASH-tagged nuclei could mask the beads, and lower the background comparing to that from untagged samples. We describe this in the Method section (l.512-519).

5. It would be great to have some 'enrichment' assessment for the overlap of CRTC-H3K9ac-H4K16ac (using FDRs as described above). The authors can use a permutation-based approach or hypergeometric test to estimate if this overlap is non-random.

As the reviewer recommended, we examined the overlaps by hypergeometric test, phyper in R. The results are presented in Fig. 3k and 3l by asterisks ($p<0.01$), indicating that most of the overlaps are not random, except the overlap of decrease in H4K16Ac and CREB/CRTC. As the reviewer expected, this will add the relevance of targeting the overlapped genes.

6. I am really not sold on the GO analysis in Fig.3i. Showing three terms, two of which have not much to do with learning processes is not very convincing. The figure is also not very appealing but this is a negligible problem.

The reviewer commented that the results of our GO analysis in the previous manuscript did not seem informative. Although we removed the data from the main figure, we showed a new list of GO-enriched term in Sup. Table 8-12, obtained from the new gene lists. The functional clustering based on the relationship between the GO-terms mostly showed the enrichment of GO-terms directly related to the neural functions (Sup. Table 8).

7. What is the rationale for only looking at genes with increased acetylation upon learning. Wouldn't the authors miss out on all negative regulators, including e.g. repressors and miRNAs? Why disregard this information?

In the previous manuscript, we did not investigate the decrease in histone acetylation because we were biased by the idea that, due to the small population of the memory-responsible neurons in the MBs during olfactory learning (6 ± 5% MB neurons responds to an odor), the detection of the memory-related decrease in histone acetylation may be masked by majority of MB neurons that are not responsible to memory processes. However, as the reviewer expected, we found statistically significant decreases in H3K9Ac and H4K16Ac using DESeq2 (Fig. 3l, Sup. Table 10, 12). These reduction could be induced nonspecifically in whole MB neurons by the LTM protocol. Although we did not further investigate functional significance of these reduction in histone acetylation, we mentioned their possible role in LTM maintenance in Discussion (l.381-384). We would like to examine whether these reductions also contribute to LTM maintenance in the future study.

8. Would be great if the authors could recheck their numbers. I just quickly checked the number of CREB/CRTC genes (1396 instead of 1394) and the ones with increasing H3K9ac (348 entries instead of 346). Seems like a systematic error... (cat | cut -f4 | sort -u | wc -l)

We have checked number of genes and provide the new gene lists in Supplemental Tables.

9. In the section 'Epigenetically modified target genes are required for early LTM maintenance' the authors short-list 20 genes, all epigenetic regulators and other 'representative genes'. Could you please explain how you got to these 'representative genes', as I couldn't find an explanation in the material and methods section. Also, isn't it interesting that none of the selected chromatin modifiers showed any impact on LTM maintenance?

The reviewer questioned how we selected 'representative genes' in the 20 genes, which we didn't fully explain in the previous manuscript. This was because we started behavioral examination based on the very initial pilot experiment, and we had to refine the gene list according to the data processing. From the initial pilot experiment without biological replicates, we obtained total 50 candidate genes. To confirm that we focused on positive hits, we tested almost all candidate's RNAi in behavioral memory test, at the same time, we added the biological replicates of ChIP-seq for histone acetylation. Because 20 out of 50 genes showed significant change in histone acetylation, without an FDR estimation, though, the data of 20 genes were presented in the previous manuscript. It was now downscaled to 10 genes by proper FDR estimation using the DESeq2 package. We believe that analysis using biological replicates and by proper FDR estimation significantly increased the validity and the reproducibility of our work. These process to select the candidate genes were explained in the Method section (l.648-654).

The reviewer also mentioned the data in which the selected chromatin modifiers didn't have any role in LTM maintenance. There are nearly 50 of chromatin modifiers in flies, which may compensate the functions of the selected chromatin modifiers (l.372-375).

10. I am more than a little intrigued by the finding that CREB/CRTC target genes show acetylation changes in a memory formation paradigm (Fig. 4b and c). CRTC, Tip60, and Gcn5 have no effect on memory formation (Fig. 1) yet they change the acetylation and polII occupancy of memory maintenance target genes in a memory formation paradigm. Could you please also compare the PolII, CRTC, and acetylation levels to the naïve state? Along the lines, why does Fig. 4F only contain Smr levels?

The reviewer asked whether PolII binding, CRTC binding and histone acetylation in the naïve state can be affected by knockdown of *CRTC*, *Tip60* and *GCN5*. Although knockdown of *CRTC* or *Tip60* didn't affect,

knockdown of *GCN5* resulted in decreased binding of PolII S2P at *Bx* and *Smr*, and also CRTC binding at *Bx* in naïve flies (Sup. Fig. 10a, b). Interestingly, H3K9Ac was not affected by knockdown of *GCN5* (Sup. Fig. 10a). This suggests that GCN5 has a structural role other than its HAT activity in naïve flies, which could be mediated by a GCN5-containing complex, SAGA. Spaced training may function to recruit Tip60 and CRTC, and also induce GCN5 HAT activity. We have revised the text to include these results accordingly (l.258-265).

The reviewer also questioned why H4K16Ac was tested only on *Smr* in Fig. 5d (in the previous figure, Fig. 4F). This is because our ChIP-seq analysis determined increase in H4K16Ac at *Smr*, but not other genes involved in 4-day LTM maintenance. We mentioned this in the text (l.241-242).

Finally, the reviewer raised an issue, in which CRTC, Tip60 and GCN5 are not important for 1d memory performance (Fig. 1b), although they are required for PolII binding and for histone acetylation to the memory maintenance genes (Fig. 5c and 5d). Importantly, the genes regulated by CRTC are LTM maintenance genes (*Bx* and *Smr*), which are not required for 1-day memory. (Fig. 4e). These results strongly suggest that ongoing transcription at 1 d after spaced training is not required for the memory performance at the same day, but is required for the memory performance at the following day such as 4 d. Thus, the time of transcription and its related memory performance could be dissociated. We incorporated the above notion in Result (l.254-257).

.

11. It would be great if the authors re-word some of their statements. For one, the authors use the word 'epigenetic regulators' to specify a set of 'chromatin modifiers'. Epigenetic regulation far exceeds chromatin modifications (e.g. miRNAs...). I would also like to stress the point that HATs do have other targets than histones, among them many transcription factors (e.g. Gcn5). Attributing LTM performance changes purely to possible histone PTM changes is a very simplistic view. A nice treatment of this fact can be found in the above-mentioned review by Barco (PMID: 25269450).

As the reviewer suggested, we thus re-worded ‘epigenetic regulators’ to ‘chromatin modifiers’. The reviewer also mentioned that HATs have additional targets to histones. This is an important caveat in this field. As the reviewer suggested, we referred the review article written by Lopez-Atalaya and Barco (2014), and included this possibility in Discussion (l.387-389).

<u>Reviewers' comments</u>:

Reviewer #2 (Remarks to the Author):

The authors have conducted an exhaustive revision of the manuscript, including numerous new experiments. As a result, the study is very much improved and convincing.

Comment on Ref #1 report:

As concluded in my second report, the authors have added an impressive amount of new results and controls to the revised manuscript. I think that they have also addressed most of the concerns expressed by Reviewer #1. I found the reply to points 1 (although I should note that I am not not an expert in Drosophila genetics and neuroanatomy), 7 and 9 satisfactory. reagrding pt. 2, as indicated by the authors (and Referee #1), the distinction between retrieval and maintenance is not possible, but the new text clarifies this point and leaves the two options open.

Reviewer #3 (Remarks to the Author):

In general, I think the authors have done a good job addressing the reviewers concerns and comments. The research results are still of high relevance to the community and the changes have definitely improved the overall quality of the manuscript. Unfortunately, I still have a couple of concerns which I deem relevant:

1. The authors show in the vennDiagram of Fig. 3k and l overlaps of CREB/CRTC-binding genes where CRTC binding is increased, altogether 2831 genes. If CRTC is a transcriptional activator of memory genes how come that half of the observed histone acetylation changes go down in response (and H3K9ac and H3K16ac are also thought to be activity marks)? How does gene expression and PolII data fit into this picture? I find it somewhat surprising that the authors have not included at least a comment that would address this 'issue'. Again, the story is to a large degree centered on CRTC as a necessary activator of gene expression, yet the histone modification data does not necessarily support this.

2. I honestly don't understand Fig. 3 h and i. First of all, H3K9ac and H4K16ac are marks known to be strongly enriched at promoter sites, decaying more or less strongly into the gene body. It is therefore neither surprising nor particularly informative to highlight this fact. Moreover, and more intriguingly to me, how can you plot a frequency of enriched regions around TSS? What is that frequency referring to and why is it not enough to give the numbers of up- and down-regulated histone marks at TSS regions (as you have done)?

3. Along the lines, given that the number of down-regulated histone acetylations in all cases exceeds the number of up-regulated cases it is even more surprising why the authors have not analyzed the role of these genes in more detail. This is also in light of the observed

discrepancies with the increased CRTC binding and the in cases down-regulated histone acetylations.

4. The y-axis in browser tracks (e.g. Fig. 3 e and j, Suppl. Fig. 8) need to be labelled. Also, it is very important to scale browser profiles to their library size (or use some other normalization that takes account of changes in total read numbers). Since the authors have not commented on any kind of normalization I suppose they have taken unadjusted values. This needs to be addressed and properly corrected.

5. In my personal opinion, supplementary Figures 5 and 6 should be discarded. I don't think Fastqc per-base-quality plots should go into the supplementary figures. I would rather suggest keeping average per-base-quality values in a table.

6. The tables of up and down-regulated histone modifications, for the same histone modifications, should be combined.

7. I am not altogether happy with how the authors have addressed one of my comments (see exchange below):

I'll have to start with another 'unfortunately', as parts of the novelty of the manuscript has been addressed in a recent study (PMID: 26656643). Especially the part of changes in regulatory networks between formation and LTM is described and should be adequately cited. In consequence, many parts of the manuscript need to be reformulated, from the summary to the discussion. Again, this is not the authors fault but still needs to be addressed appropriately.

As the reviewer suggested, we cite the work conducted by Halder et al (2016) (PMID: 26656643). In particular, Halder et al reported that, in mice, DNA methylation is a prominent indicator of gene expression related to memory, but various histone modifications, including H3K9Ac, are rather decoupled to gene expression. Interestingly, our results suggest that histone acetylation could be coupled to gene expression in flies. We think that such species differences would be reasonably explained by the fact that flies lack DNA methylation mechanisms and therefore histone acetylation in flies could have much more significant roles in gene expression. We have included these discussion in the text (l.376-381).

The authors now write 'However, studies in other organisms suggest that DNA methylation, but not histone acetylation, correlates to gene expression16.'. This sentence is not correct, the referenced article doesn't state that histone acetylation doesn't correlate with gene expression. It rather states that the correlation with gene expression is surprisingly low, whereas the correlation with DNA methylation seems to be higher. I would also like to highlight that the article covers changes in gene activity programs between memory formation and LTM (as mentioned in the initial comment). The authors write that 'However, it has been unclear whether epigenetic alterations during maintenance simply extend transcriptional changes induced during formation, or whether they induce novel transcriptional changes.'. Again, this part has been addressed in PMID:26656643, albeit not

to the same extend as in this manuscript but it still deserves recognition.

8. Finally and least importantly, the artwork in Fig. 8 could be improved. Why are the chromatin binding complexes tilted?

Comment on Ref #1's concerns:

Unfortunately, the reviewers concerns were addressed only in part. Especially the first two points could have been addressed better with simple changes to the text but the authors either failed or ignored to do so. I still think this research should be published as it gives important insights to memory maintenance/retrieval in flies but the manuscript in its current state still needs a thorough work-over. In the following paragraphs I address points 1, 2, and 7 that were raised by reviewer 1 in more detail:

pt. 1) The reviewer states that changes in transcriptional machinery could be attributed to different subpopulations of Kenyon cells that are active at different time-points during the experiments. Although surely a point of consideration changes in subpopulations would not negate the observed changes in transcriptional machiner but would merely require an addition to the text. In the revised manuscript, the authors tried to address this with further experiments although the results are not finally conclusive. Nevertheless, the authors mention in the revised version of the manuscript that the possibility exists that different cell types are responsible for the observed shifts in the transcriptional machinery 'Although we propose that the shifts in transcriptional regulation we observe occur temporally in the same cells, we cannot discount the possibility that LTM lasting 7 days is maintained in different cells from LTM lasting 4 days. MB Kenyon cells can be
separated into different cell types, which exert differential effects on learning,
short-term memory, and LTM. Thus it is possible that LTM itself consists of different
types of memory that can be separated anatomically. In this case, CRTC in one cell type
may exert non-direct effects on another cell type to activate downstream genes
including Bx and Smr. However, given that CRTC binds to the Bx gene locus to
promote Bx expression, and both CRTC and Bx were required in the same α/βs subtype
of Kenyon cells, we favor a simpler model where CRTC binding, histone acetylation,
and downstream gene induction occur in the same cells.'. I think this point is addressed reasonably well, although I am not a big fan of purely subjective statements such as 'we favor a simpler model where CRTC binding, histone acetylation, and downstream gene induction occur in the same cells'.

pt. 2) The reviewer highlighted the problem that the reduced memory performance could be explained by impaired memory retrieval and might not be an effect of impaired memory maintenance. This is a very valid point and although the authors have addressed this issue in their discussion 'Currently, we propose that the alterations in histone acetylation and transcription we
uncovered are required for memory maintenance. However, we note that decreases in

memory after formation could be caused by defects in retrieval as well as maintenance. Thus, if recall of LTM requires changes in transcription, and if recall at different times after training require different transcriptional changes, it remains possible that the epigenetic and transcriptional changes we found are required for recall and not maintenance.' The authors literally state that 'decreases in memory after formation could be caused by defects in retrieval as well as maintenance' and I do not understand why they haven't changed the manuscript accordingly. As the authors are unable to disambiguate between retrieval and maintenance they should avoid statements such as 'Shifting transcriptional machinery is required for long-term memory maintenance and modification in Drosophila mushroom bodies' in the title of the manuscript. In consequence, I personally think this issue needs more thorough attention and I wish the authors would have put some more effort into it in the resubmission of their manuscript.

pt. 7) The reviewer complained about the quality of the images that show 'nuclear translocation of HA-tagged CRTC' as the quality of the images is not sufficient to the this transition. The authors have supplied a new set of images that should show this translocation in Fig. 1 and Sup. Fig. 1. I have to admit that although Fig. 1 panels d,g, and j provide high-quality images of nuclear CRTC-HA the panels e,h, and k of Fig. 1 seemt to be out of focus (blurry, artifact by the projection method?) and do not necessarily substantiate the claims of nuclear translocation. I should however mention that the observed changes are rather small (~30%) and images might not reflect these changes appropriately.

Responses to Reviewer #3:

We thank again the comments from Reviewer #3. In our revised manuscript, we addressed all the issues by adding new data and modifying the text.

Our point to point responses are as follows.

1. The authors show in the Venn Diagram of Fig. 3k and l overlaps of CREB/CRTC-binding genes where CRTC binding is increased, altogether 2831 genes. If CRTC is a transcriptional activator of memory genes how come that half of the observed histone acetylation changes go down in response (and H3K9ac and H3K16ac are also thought to be activity marks)? How does gene expression and PolII data fit into this picture? I find it somewhat surprising that the authors have not included at least a comment that would address this 'issue'. Again, the story is to a large degree centered on CRTC as a necessary activator of gene expression, yet the histone modification data does not necessarily support this.

Given that CRTC is a transcriptional activator, CRTC binding is predicted to overlap with increase in histone acetylation. However, some of the CRTC target genes showed decrease in histone acetylation, and therefore the reviewer asked for a comment on this issue. As mentioned in the revised manuscript (Discussion, l. 382-384), we predict that the transcriptional repressors are involved in the reductions of histone acetylation. Although yet unidentified, those putative repressors may interact with CREB2b/CRTC, thereby reduce histone acetylation. CREB2b is the repressor isoform of CREB that binds to CRE site and still carries the interaction domain with CRTC. Alternatively, other transcriptional repressors may bind nearby CREB/CRTC, thereby decrease histone acetylations. Since either model is still highly speculative, and its impact on gene expression is currently unclear, in this paper we focused on the transcriptional activation through CRTC and histone acetylation. We would like to consider decrease in histone acetylations and the aforementioned putative repressors as a future research subject.

2. I honestly don't understand Fig. 3 h and i. First of all, H3K9ac and H4K16ac are marks known to be strongly enriched at promoter sites, decaying more or less strongly into the gene body. It is therefore neither surprising nor particularly informative to highlight this fact. Moreover, and more intriguingly to me, how can you plot a frequency of enriched regions around TSS? What is that frequency referring to and why is it not enough to give the numbers of up- and down-regulated histone marks at TSS

regions (as you have done)?

In the previous manuscript, Fig. 3h and i were intended to show the soundness of our analysis, by showing that alteration of histone acetylation occurred nearby TSS over a range of tens of Kb, because, as the reviewer mentioned, histone acetylation is well-known to be enriched near TSS. “Frequency” of the ordinate of the panels was defined as follows. The numbers of the increased- or the decreased-histone acetylation sites were counted in 200 bp bins nearby TSS, and each number was divided by the total number. However, in the previous revised manuscript, we have already added the alternative data, the aggregate plot (Fig. 3f and g), showing the enrichment of histone acetylation nearby TSS, and also mentioned in the text (l. 179-182, 184-187) that the majority of the histone acetylation change occurs in the vicinity of TSS. As the reviewer pointed out, these would be enough to add the relevance of our study. We therefore omitted the previous Fig. 3h and i.

3. Along the lines, given that the number of down-regulated histone acetylations in all cases exceeds the number of up-regulated cases it is even more surprising why the authors have not analyzed the role of these genes in more detail. This is also in light of the observed discrepancies with the increased CRTC binding and the in cases down-regulated histone acetylations.

We agree with the reviewer, regarding the genes with decrease in histone acetylations, which would be of interest to investigate. As we mentioned in the response #1, the decreased histone acetylations are only explained by the transcriptional repression. Given that our manuscript is focused on the upregulation of the gene expression through CREB/CRTC and histone acetylations, we believe that testing the down-regulated genes by the repressors, is not part of our current study. However, as we think the reviewer’s comment plausible, we mentioned the possible significance of the down-regulated genes (Discussion, l. 384-388).

4. The y-axis in browser tracks (e.g. Fig. 3 e and j, Suppl. Fig. 8) need to be labelled. Also, it is very important to scale browser profiles to their library size (or use some other normalization that takes account of changes in total read numbers). Since the authors have not commented on any kind of normalization I suppose they have taken unadjusted values. This needs to be addressed and properly corrected.

As the reviewer pointed out, we have corrected and labeled the y-axis in Fig. 3e, j and Suppl. Fig. 8 (in the current manuscript, Fig. 3e, h, and Suppl. Fig. 6), which were properly adjusted by their library size.

5. In my personal opinion, supplementary Figures 5 and 6 should be discarded. I don't think Fastqc per-base-quality plots should go into the supplementary figures. I would rather suggest keeping average per-base-quality values in a table.

We follow the reviewer's recommendation to omit the previous Sup. Fig. 5 and 6, and left the average per-base-quality values in Sup. Table 1.

6. The tables of up and down-regulated histone modifications, for the same histone modifications, should be combined.

As the reviewer suggested, the tables indicating the same histone modifications were combined; Sup. Table 3 (increase in H3K9Ac) and 5 (decrease in H3K9Ac), Table 4 (increase in H4K16Ac) and 6 (decrease in H4K16Ac)

7. I am not altogether happy with how the authors have addressed one of my comments (see exchange below):

I'll have to start with another 'unfortunately', as parts of the novelty of the manuscript has been addressed in a recent study (PMID: 26656643). Especially the part of changes in regulatory networks between formation and LTM is described and should be adequately cited. In consequence, many parts of the manuscript need to be reformulated, from the summary to the discussion. Again, this is not the authors fault but still needs to be addressed appropriately.

As the reviewer suggested, we cite the work conducted by Halder et al (2016) (PMID: 26656643). In particular, Halder et al reported that, in mice, DNA methylation is a prominent indicator of gene expression related to memory, but various histone modifications, including H3K9Ac, are rather decoupled to gene expression. Interestingly, our results suggest that histone acetylation could be coupled to gene expression in flies. We think that such species differences would be reasonably explained by the fact that flies lack DNA methylation mechanisms and therefore histone acetylation in flies could have much more significant roles in gene expression. We have

included these discussion in the text (l.376-381).

The authors now write 'However, studies in other organisms suggest that DNA methylation, but not histone acetylation, correlates to gene expression16.'. This sentence is not correct, the referenced article doesn't state that histone acetylation doesn't correlate with gene expression. It rather states that the correlation with gene expression is surprisingly low, whereas the correlation with DNA methylation seems to be higher. I would also like to highlight that the article covers changes in gene activity programs between memory formation and LTM (as mentioned in the initial comment). The authors write that 'However, it has been unclear whether epigenetic alterations during maintenance simply extend transcriptional changes induced during formation, or whether they induce novel transcriptional changes.'. Again, this part has been addressed in PMID:26656643, albeit not to the same extend as in this manuscript but it still deserves recognition.

As suggested by the reviewer, we corrected our inappropriate descriptions for the previous work by Halder et al (2016) (PMID: 26656643), and modified the text in introduction and discussion. The followings are the examples:

In Introduction,
“In mice, a recent study reported alterations in DNA methylation coupled to gene expression after learning, and importantly, this study demonstrated differences in gene expression between LTM formation and maintenance” (l. 60-63)

In Discussion,
“DNA methylation shows higher correlation to gene expression comparing to histone acetylation in mice” (l. 377-378)
“Consistent to the previous study in mice, which suggests distinct transcriptional regulations in LTM formation and maintenance, our data indicate that memory formation and maintenance are distinct processes.” (l. 363-365)

8. Finally and least importantly, the artwork in Fig. 8 could be improved. Why are the chromatin binding complexes tilted?

As the reviewer suggested, Fig. 8 have been amended and now all labels for the chromatin binding complexes become straight.

Comment on Ref #1's concerns:

Unfortunately, the reviewers concerns were addressed only in part. Especially the first two points could have been addressed better with simple changes to the text but the authors either failed or ignored to do so. I still think this research should be published as it gives important insights to memory maintenance/retrieval in flies but the manuscript in its current state still needs a thorough work-over. In the following paragraphs I address points 1, 2, and 7 that were raised by reviewer 1 in more detail:

pt. 1) The reviewer states that changes in transcriptional machinery could be attributed to different subpopulations of Kenyon cells that are active at different time-points during the experiments. Although surely a point of consideration changes in subpopulations would not negate the observed changes in transcriptional machiner but would merely require an addition to the text. In the revised manuscript, the authors tried to address this with further experiments although the results are not finally conclusive. Nevertheless, the authors mention in the revised version of the manuscript that the possibility exists that different cell types are responsible for the observed shifts in the transcriptional machinery
'Although we propose that the shifts in transcriptional regulation we observe occur temporally in the same cells, we cannot discount the possibility that LTM lasting 7 days is maintained in different cells from LTM lasting 4 days. MB Kenyon cells can be separated into different cell types, which exert differential effects on learning, short-term memory, and LTM. Thus it is possible that LTM itself consists of different types of memory that can be separated anatomically. In this case, CRTC in one cell type may exert non-direct effects on another cell type to activate downstream genes including Bx and Smr. However, given that CRTC binds to the Bx gene locus to promote Bx expression, and both CRTC and Bx were required in the same α/βs subtype of Kenyon cells, we favor a simpler model where CRTC binding, histone acetylation, and downstream gene induction occur in the same cells.'
I think this point is addressed reasonably well, although I am not a big fan of purely subjective statements such as 'we favor a simpler model where CRTC binding, histone acetylation, and downstream gene induction occur in the same cells'.
We withdrew the subjective wording, which was pointed out by the reviewer, “we favor a simpler model where CRTC binding, histone acetylation, and downstream gene induction occur in the same cells”. We instead finished the paragraph by mentioning the

solid interpretation from our data: “However, since that CRTC binds to the Bx gene locus to promote Bx expression, and both CRTC and Bx are required in the same α/βs subtype of Kenyon cells, it is likely that the shift from CRTC-dependent to Bx-dependent transcription occurs within the α/βs neurons” (l. 350-353).

pt. 2) The reviewer highlighted the problem that the reduced memory performance could be explained by impaired memory retrieval and might not be an effect of impaired memory maintenance. This is a very valid point and although the authors have addressed this issue in their discussion
'Currently, we propose that the alterations in histone acetylation and transcription we uncovered are required for memory maintenance. However, we note that decreases in memory after formation could be caused by defects in retrieval as well as maintenance. Thus, if recall of LTM requires changes in transcription, and if recall at different times after training require different transcriptional changes, it remains possible that the epigenetic and transcriptional changes we found are required for recall and not maintenance.'
The authors literally state that 'decreases in memory after formation could be caused by defects in retrieval as well as maintenance' and I do not understand why they haven't changed the manuscript accordingly. As the authors are unable to disambiguate between retrieval and maintenance they should avoid statements such as 'Shifting transcriptional machinery is required for long-term memory maintenance and modification in Drosophila mushroom bodies' in the title of the manuscript. In consequence, I personally think this issue needs more thorough attention and I wish the authors would have put some more effort into it in the resubmission of their manuscript.

We agree with the reviewer’s criticism that, although it was not conclusively demonstrated whether the transcriptional regulation is required for LTM maintenance or LTM retrieval, the previous manuscript argued the former. We now added new data, indicating that CREB/CRTC is indeed important for LTM maintenance: overexpression of CREB2b or knockdown of CRTC 1-4 day after spaced training impaired 7-day memory (Fig. 1c). If CREB/CRTC is not required for LTM maintenance, this temporal manipulation doesn’t affect the 7-day memory. Because we believe that the role of CREB/CRTC in LTM maintenance is now firmly demonstrated, we left the title as before. However, their roles in LTM retrieval is currently unknown, because acute inhibition of CREB/CRTC at the time of memory retrieval is experimentally difficult to

perform. Therefore, in Discussion, we left the previous comment on this possibility as follows (l. 355-356).
“However, we note that decreases in memory after formation could be caused by defects in retrieval as well as maintenance.”

pt. 7) The reviewer complained about the quality of the images that show 'nuclear translocation of HA-tagged CRTC' as the quality of the images is not sufficient to the this transition. The authors have supplied a new set of images that should show this translocation in Fig. 1 and Sup. Fig. 1. I have to admit that although Fig. 1 panels d,g, and j provide high-quality images of nuclear CRTC-HA the panels e,h, and k of Fig. 1 seemt to be out of focus (blurry, artifact by the projection method?) and do not necessarily substantiate the claims of nuclear translocation. I should however mention that the observed changes are rather small (~30%) and images might not reflect these changes appropriately.

As pointed out by the reviewer, we improved the high-magnification panel showing the nuclear translocation of CRTC-HA (Fig. 1 and Sup. Fig. 1). We subjected the confocal series of the images to the deconvolution software and obtained the clearer pictures. The reviewer also questioned whether those magnified pictures reflect the 30% increase in the median nuclear CRTC-HA (as appeared in Fig. 1g). However, the magnified pictures are the clipped images showing the robust nuclear CRTC translocation occurred in 4% of the neurons, at the level of the dendritic region, as shown in Fig. 1n. Therefore, as the reviewer noticed, it merely reflect the 30% increase in the median nuclear CRTC-HA.

REVIEWERS' COMMENTS:

Reviewer #3 (Remarks to the Author):

I think the article is now ready for publication.
